# Global thyroid cancer incidence in relation to dietary and socioeconomic factors: an ecological machine learning study

**DOI:** 10.3389/fonc.2026.1842508

**Published:** 2026-10-16

**Authors:** Yixing Li, Dehui Liu, Ruixue Qin, Xingchang Li, Mei Li

**Affiliations:** 1 The First College of Clinical Medical Science, China Three Gorges University; Yichang Central People's Hospital, Yichang, Hubei, China; 2 Department of Oncology, Yiling Hospital of Yichang, Yichang, Hubei, China

**Keywords:** ecological study, GLOBOCAN, FAOSTAT, iodine-rich food availability, machine learning, path decomposition, seafood, thyroid cancer

## Abstract

**Background:**

Thyroid cancer incidence has risen dramatically worldwide, with marked geographic heterogeneity suggesting dietary and environmental contributions beyond screening effects. The roles of specific dietary patterns in thyroid cancer remain poorly characterized at the global level.

**Objectives:**

Age-standardized thyroid cancer incidence was the prespecified primary outcome. We aimed to (1) build machine learning models predicting country-level thyroid cancer incidence from dietary and socioeconomic features, (2) quantify confounder-adjusted dietary associations with incidence, including prespecified sensitivity analyses of the iodine-rich food availability index, and (3) identify key dietary predictors through explainability analyses and a descriptive decomposition of the development pathway. Mortality was analyzed only as a secondary outcome in order to evaluate whether the incidence patterns could be attributed to differential cancer detection rather than to differences in disease occurrence.

**Methods:**

We integrated GLOBOCAN 2022 (cancer outcomes), FAOSTAT Food Balance Sheets (113 food items, 2010–2022; primary exposure 2015–2019 mean), the UNDP Human Development Report 2025 statistical annex (HDI 2023 and its life expectancy, education, and income sub-indices), and WHO obesity prevalence (2022) across 163 countries, yielding 155 harmonized variables and 150 numeric predictors. All modeling was performed inside a nested cross-validation framework: multiple imputation by chained equations, standardization, four-method consensus feature selection, and hyperparameter search were fitted exclusively on the training partition of each outer fold (10 folds × five repetitions), and performance was summarized from pooled out-of-fold predictions with 95% confidence intervals obtained by resampling countries (2,000 bootstrap replicates). Three analyses separated dietary signal from confounding: block-wise models comparing socioeconomic-only, diet-only and combined feature sets; leave-one-region-out validation across six world regions; and confounder-adjusted partial correlations under a basic (HDI, obesity) and an extended adjustment set (HDI, obesity, the three HDI sub-indices, and region fixed effects), with Benjamini–Hochberg and Bonferroni correction applied across all comparisons. Iodine-related exposure was represented by an iodine-rich food availability index constructed from seafood, dairy, and egg supply; its construction was interrogated in four sensitivity analyses in which the components were entered separately and jointly, four weighting schemes were compared, total animal protein supply was added to the adjustment set, and an iodine-poor animal-origin food index was used as a specificity control. Binary classification and all mortality analyses were secondary.

**Results:**

Regression on incidence was the primary task. Stacking ensemble performed best, with a pooled out-of-fold *R*² of 0.613 (95% CI [0.510, 0.726]), RMSE = 3.04, MAE = 1.82, and Spearman *ρ* = 0.901 (95% CI [0.862, 0.924]); random forest (*R*² = 0.609), bagging (*R*² = 0.599), and KNN (*R*² = 0.580) followed, and a high-dimensional deep tabular model (DynaTab) was statistically comparable (*R*² = 0.626). Predictive accuracy was, however, almost entirely socioeconomic: nine development variables alone gave *R*² = 0.609, 140 dietary variables alone gave 0.284, and the incremental contribution of the dietary block was –0.048 (95% CI −0.169 to +0.034), +0.021, and +0.017 across model families. Under leave-one-region-out validation, the rank ordering of countries was largely preserved (pooled *ρ* = 0.813), but the level was not. Unadjusted associations with incidence were positive for the iodine-rich food availability index (*ρ* = 0.234), total energy (*ρ* = 0.230), seafood (*ρ* = 0.219), and the animal-to-plant protein ratio (*ρ* = 0.221). After adjustment and Benjamini–Hochberg correction, seafood supply, dairy supply, the iodine-rich food availability index, the animal-to-plant protein ratio, and mollusks remained significant under the extended adjustment set (seafood partial *r* = 0.196, *q* = 0.031; iodine-rich food availability index 0.224, *q* = 0.026), whereas plant protein did not (–0.095, *q* = 0.309). In sensitivity analyses, the index association was carried by seafood (0.196, *q* = 0.017) and dairy (0.198, *q* = 0.017) but not eggs (0.065, *q* = 0.411), was stable across equal-weighted, z-score, and iodine-content-weighted constructions (0.238–0.256), survived further adjustment for total animal protein supply (0.203, *q* = 0.032), and was not reproduced by an iodine-poor animal-origin food index (0.102, *q* = 0.198). Path decomposition attributed 50.2% of the seafood–incidence correlation to the HDI pathway (95% bootstrap CI 16%–90%). In the secondary mortality analysis, the dietary ordering was preserved, but associations with the mortality-to-incidence ratio were close to zero (HDI *ρ* = 0.053; seafood *ρ* = 0.065), and in the sensitivity classification analysis, Extra Trees reached AUC = 0.932 (95% CI [0.893, 0.964]) at the median split.

**Conclusions:**

With all model construction confined to training folds, machine learning models built from national food supply and socioeconomic data explain a moderate share of the between-country variation in thyroid cancer incidence, but that predictive accuracy is carried by development indicators rather than by diet. The availability of iodine-rich food groups, seafood supply, and the animal-to-plant protein ratio nevertheless retained positive ecological associations with incidence after adjustment for development and metabolic confounders and after correction for multiple comparisons, and the index association proved robust to how the index was constructed and specific to iodine-rich rather than to animal-origin food groups in general; plant protein showed no association once multiplicity was controlled. Roughly half of the seafood–incidence correlation tracks the development pathway, and the secondary mortality analysis is consistent with a substantial contribution from differential detection. These findings are ecological associations with the availability of iodine-rich food groups, not with measured iodine intake or exposure; they generate testable hypotheses for individual-level studies with measured iodine status and do not establish causation.

## Highlights

- GLOBOCAN 2022 thyroid cancer estimates were integrated with FAOSTAT food balance sheets, UNDP Human Development Report 2025 indices, and WHO obesity prevalence across 163 countries, yielding 155 harmonized variables and a 150-variable modeling matrix. Age-standardized incidence was the prespecified primary outcome; mortality was examined only as a secondary analysis of differential cancer detection.
- A total of 13 regression algorithms were evaluated inside a fully nested cross-validation design in which imputation, standardization, four-method feature selection, and hyperparameter tuning were refitted within every training fold so that no held-out country influenced model construction; seven classifiers were evaluated under the same design but retained only as a sensitivity analysis.
- The best regression model (stacking ensemble) reached a pooled out-of-fold *R*² of 0.613 (95% country-level bootstrap CI [0.510, 0.726]) with Spearman *ρ* = 0.901 between predicted and observed rates; ensembles outperformed regularized linear models only modestly (Lasso *R*² = 0.579) and unregularized linear regression clearly (*R*² = 0.312). A high-dimensional deep tabular model (DynaTab), benchmarked under the same nested protocol, was statistically comparable (pooled *R*² = 0.626; Section 4.2.1).
- Block-wise models showed that this predictive accuracy is socioeconomic rather than dietary: nine development variables alone reached *R*² = 0.609, 140 dietary variables alone reached 0.284, and adding the dietary block to the development block changed *R*² by –0.048, +0.021, and +0.017 across three model families, none of which excluded zero.
- Under the extended adjustment set (HDI, its three sub-indices, obesity prevalence, and region fixed effects) with Benjamini–Hochberg correction across all comparisons, seafood supply, dairy supply, the iodine-rich food availability index, the animal-to-plant protein ratio, and mollusks retained positive associations with incidence (for example, seafood partial *r* = 0.196, *q* = 0.031; iodine-rich food availability index 0.224, *q* = 0.026), whereas plant protein did not (–0.095, *q* = 0.309); a descriptive path decomposition attributed 50.2% (95% bootstrap CI 16%–90%) of the seafood–incidence correlation to the HDI pathway.
- Prespecified sensitivity analyses of the iodine-rich food availability index showed that its association with incidence is carried by seafood and dairy availability rather than by eggs, is insensitive to how the three components are weighted (extended-adjustment partial *r* = 0.196–0.256 across four alternative constructions), persists after further adjustment for total animal protein supply (0.203, *q* = 0.032), and is not reproduced by an iodine-poor animal-origin food index (0.102, *q* = 0.198).

## 1 Introduction

Thyroid cancer is the most common endocrine malignancy worldwide (1). In 2022, 821,214 new cases of thyroid cancer were recorded, and 47,616 people died from the disease (2, 3). The global age-standardized incidence rate of thyroid cancer has increased by 3% to 4% per year over the past three decades, a rise that is much greater than can be explained by any single pathophysiological mechanism (4). Approximately 85% of all cases are classified as papillary thyroid carcinoma (PTC), which has a good prognosis but is still associated with considerable burden of diagnostic, treatment-related, and psychosocial cost (5). It is urgent that a public health response to the forces contributing to this increase globally be established.

Incidence rates of thyroid cancer vary between sub-Saharan African countries where rates may be less than one per 100,000 to rates exceeding 25–30 per 100,000 in countries such as South Korea, Cyprus, and French Polynesia, with a disparity of more than 30-fold (6). Part of the reason for these differences in incidence can be explained by access to ultrasonography; South Korea’s dramatic reduction in incidence after changing its screening recommendations shows how much of the increase was due to overdiagnosis (7, 8). Even so, there are still differences in rates between countries that have a similar level of access to screening, suggesting that there are environmental and dietary factors which are contributors independently but that have not been adequately characterized at a global level (9).

Among environmental factors hypothesized to contribute to thyroid cancer development, dietary iodine plays a physiological role in thyroid cell function. Chronic TSH elevation due to low iodine availability has been associated with increased follicular thyroid cancer risk, while high iodine intake has been proposed to increase oxidative DNA damage, potentially elevating papillary thyroid cancer risk (10, 11). Meta-analytic evidence on iodine intake and thyroid cancer risk remains inconsistent (10). Western dietary patterns have been proposed to influence cancer risk through inflammatory and metabolic pathways, while plant-based diets have been associated with potential protective effects, possibly via isoflavones and phytochemicals (12, 13). A Mendelian randomization study has reported associations consistent with a diet–thyroid cancer relationship, though ecological and individual-level confirmation remains needed (14).

Obesity is a downstream consequence of dietary imbalance consistently associated with elevated thyroid cancer risk (15, 16). Proposed mechanisms include chronic inflammation, hyperinsulinemia, and elevated IGF-1 bioavailability. The parallel global rises in obesity and thyroid cancer incidence suggest that dietary exposures operate through multiple pathways—direct nutrient effects, metabolic consequences, and confounding by healthcare access—requiring analytical frameworks capable of simultaneously accommodating high-dimensional dietary data and socioeconomic confounding.

Structural limitations of traditional epidemiological designs are inherent in their design. While cohort studies often use one or two times to measure diet, socioeconomic status concurrently confounds dietary quality, access to healthcare, and cancer detection (9). The machine learning (ML) approach provides a framework that easily accommodates high-dimensional correlated data, captures nonlinear relationships between features, considers interactions between features, and allows for model agnostic explanations of how the model reached its outcome (17, 18). Recent ML applications have demonstrated predictive gains for colorectal and breast cancer from dietary and biomarker data (19, 20), and ML-based ecological analyses have begun identifying thyroid cancer predictors within individual countries (21, 22). Such explainable and multimodal machine learning approaches have recently proven effective across a range of complex, high-dimensional biomedical problems from single-cell and prognostic modeling in cancer (23), through multi-omics and deep-learning histopathology (24), to integrated clinical–biochemical–microbiome disease staging (25), motivating their application to the high-dimensional and collinear setting of national dietary and socioeconomic data examined here.

Ecological studies have generated foundational dietary cancer hypotheses since Armstrong and Doll (26). Despite the ecological fallacy limitation, this design is particularly informative for exposures that vary more between populations than within them—precisely the case for national food supply composition (27). The availability of comprehensive open-access databases—GLOBOCAN 2022 (2), FAOSTAT Food Balance Sheets covering 113 food items (28), UNDP Human Development Reports (29), and WHO Global Health Observatory (30)—creates an unprecedented infrastructure for large-scale ecological ML analyses integrating dietary, socioeconomic, and epidemiological dimensions.

To our knowledge, no prior ecological study has simultaneously integrated ML-based prediction, confounder-adjusted association analysis, temporal lag sensitivity across five exposure windows spanning 0–12 years, and formal HDI-pathway correlation decomposition of thyroid cancer across countries globally. Existing ML applications have been confined to single-country analyses with restricted feature sets (21, 22), and the proportion of any diet–cancer ecological association attributable to national development versus genuine dietary biology has never been formally estimated. To address these gaps, we integrated four harmonized databases to pursue three objectives, all of which take age-standardized thyroid cancer incidence as the primary outcome: (1) construct and validate ML models predicting thyroid cancer incidence from 155 engineered features using 13 regression algorithms evaluated by nested cross-validation, (2) quantify confounder-adjusted dietary associations with incidence under two confounder sets with multiplicity correction, together with prespecified sensitivity analyses of the iodine-rich food availability index and lag sensitivity across the exposure windows retained in the analytical dataset, and (3) identify key predictors via permutation importance, marginal contribution analysis, partial dependence, and a descriptive decomposition of the HDI pathway. As a secondary analysis, we examined thyroid cancer mortality and the mortality-to-incidence ratio, not as an outcome of interest in its own right but in order to evaluate whether the incidence patterns we describe could be attributed largely to differential diagnostic intensity: if incidence differences were driven only by detection, the corresponding associations with mortality would not be expected to follow. We prespecified four expectations: that ensemble algorithms would outperform regularized linear models, that the availability of iodine-rich food groups would retain a positive association after adjustment, that plant protein would show an inverse association once confounders were removed, and that a substantial share of the seafood–incidence correlation would track national development. As reported below, the second and fourth expectations were supported, the first only marginally, and the third was not supported once multiplicity was controlled.

## 2 Materials and methods

### 2.1 Study design and reporting framework

We employed a cross-sectional ecological study design using publicly available country-level datasets, linking national food supply availability indicators from FAOSTAT to national thyroid cancer incidence and mortality rates from GLOBOCAN 2022 (**Figure 1**). Age-standardized thyroid cancer incidence is the primary outcome of this study; mortality is a secondary outcome examined solely as a check on differential cancer detection (Sections 2.2.1 and 2.7.5). This design is appropriate for generating hypotheses about population-level dietary exposures that exhibit substantial between-country variation, as food balance sheet data reflect national food availability patterns rather than individual intake. We followed the Transparent Reporting of a multivariable prediction model for Individual Prognosis or Diagnosis Artificial Intelligence (TRIPOD+AI) statement where applicable (31); a completed TRIPOD+AI checklist with page references for each reporting item is provided as **Supplementary Table S1**. We also followed the Strengthening the Reporting of Observational Studies in Epidemiology (STROBE) statement for ecological studies (32), for which a completed checklist is provided as **Supplementary Table S2**.

**Figure 1:**
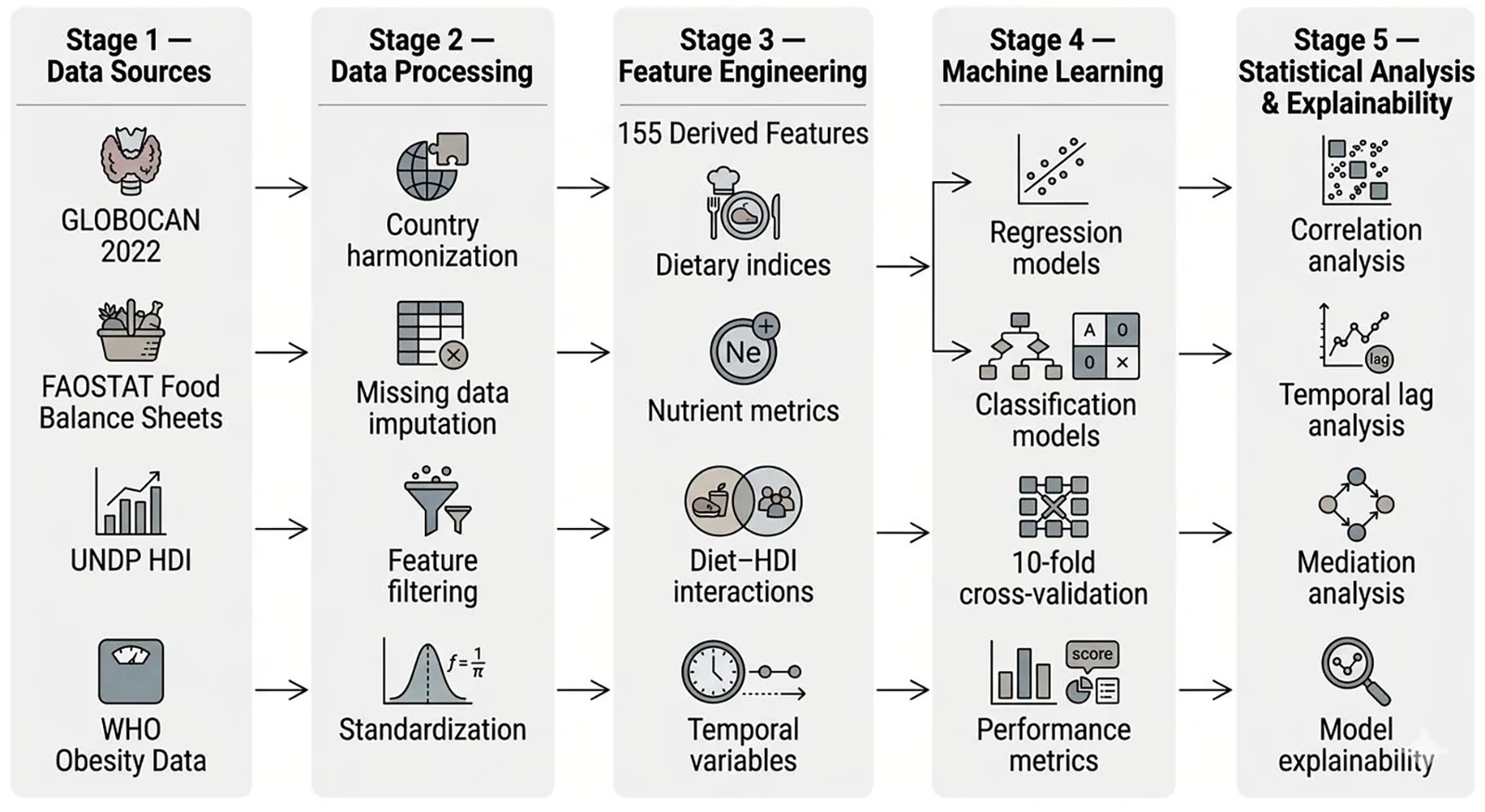
Proposed methodology framework: five-phase analytical pipeline spanning data acquisition, preprocessing (MICE imputation, M49 harmonization), feature engineering (155 variables), nested cross-validated modeling (13 regression + seven classification algorithms with all preprocessing, feature selection, and tuning performed inside training folds), evaluation with explainability (held-out permutation importance, feature contribution analysis), and statistical association analyses (multiplicity-corrected partial correlations under two confounder sets, sensitivity analyses of the iodine-rich food availability index, descriptive path decomposition, lag sensitivity, and dietary clustering). Incidence is the primary outcome throughout; mortality enters only in the secondary detection analysis.

### 2.2 Data sources and acquisition

#### 2.2.1 Thyroid cancer incidence and mortality data

Cancer incidence and mortality data were obtained from the Global Cancer Observatory (GLOBOCAN 2022) (2), maintained by the International Agency for Research on Cancer (IARC). We extracted thyroid-cancer-specific data (ICD-10 code C73) including age-standardized incidence rates (ASR, using the world standard population), crude incidence rates, cumulative risk (0–74 years), and absolute case counts for 185 countries, disaggregated by sex (0 = both sexes, 1 = male, 2 = female). The prespecified primary outcome was the combined-sex incidence ASR, given that thyroid cancer incidence is substantially higher in women (approximately 3:1 female-to-male ratio) and combined-sex ASR captures the overall population-level pattern. Every model, association analysis, and decomposition reported in Sections 3.2–3.6 takes this incidence ASR as its outcome. The mortality ASR and the mortality-to-incidence ratio were prespecified as secondary outcomes with a single, narrowly defined purpose: to assess whether the incidence patterns are attributable to differential cancer detection rather than to differences in disease occurrence (Section 2.7.5). They are reported separately in Section 3.7, and no primary conclusion of this study rests on them (2, 33). Sex-stratified Spearman correlation analyses are provided in **Supplementary Table S3** and show consistent directional findings across both sexes.

#### 2.2.2 Dietary exposure data

National food supply availability data were obtained from the FAOSTAT Food Balance Sheets (FBS) database (28). We extracted data for 113 individual food items across four nutritional dimensions: food supply quantity (kg/capita/year), energy (kcal/capita/day), protein (g/capita/day), and fat (g/capita/day). A supplementary extract of 13 seafood and dairy subcategories including freshwater fish, demersal fish, pelagic fish, crustaceans, cephalopods, mollusks, milk, and butter was assembled to capture iodine-dense food groups relevant to the exposure hypothesis. For the primary cross-sectional analyses, the mean annual food supply availability over 2015–2019 was used as the primary exposure variable, reflecting population-level dietary patterns in the years preceding the 2022 cancer outcome. Annual data from each year 2010–2022 were additionally used for temporal lag sensitivity analyses (lag 0–12 years), as described in Section 2.4.4.

#### 2.2.3 Socioeconomic and demographic confounders

Data on 2023 HDI values were obtained from **Table 1** of the statistical annex of the UNDP Human Development Report 2025 (29). The HDI column and its three sub-indices were extracted directly from that primary source and recomputed with the official HDR formulas (life expectancy index = (LE − 20)/(85 − 20); education index = the mean of expected years of schooling/18 and mean years of schooling/15, each capped at unity; income index = (ln GNI per capita − ln 100)/(ln 75,000 − ln 100)) rather than taken from secondary compilations because an internal consistency check showed that commonly circulated compilations of these values do not reproduce the published index from its own sub-indices. Our recomputed sub-indices reproduce the published HDI value to a mean absolute difference of 0.0003 across all 193 countries in the annex, and the reconstruction is released as Part 0 of **Supplementary Code S1** so that it can be verified independently. HDI was available for 161 of the 163 study countries; the two French overseas collectivities (French Polynesia and New Caledonia) are not reported in the HDI annex, and their socioeconomic values were imputed within training folds by the MICE procedure described in Section 2.3.2. The HDI is a composite index that includes (1) life expectancy at birth, (2) educational attainment measured as expected and mean years of schooling, and (3) standard of living expressed as gross national income (in 2021 PPP dollars) per person. Sub-indices for each of the HDI components were used as individual potential confounders. Obesity prevalence (prevalence of adults (both sexes) with BMI ≥30, age-standardized and as of 2022) was obtained from the WHO Global Health Observatory data set (30). HDI and obesity prevalence were used as primary confounding variables in adjusted analyses because both measures capture the primary routes of confounding: HDI measures the intensity of diagnostic testing and access to health care, and obesity measures the metabolic pathway of confounding.

**Table 1:** Regression model performance for predicting country-level thyroid cancer incidence ASR, the primary outcome (nested 10-fold × five-repetition cross-validation, pooled out-of-fold predictions, *n* = 163).

Model	Pooled out-of-fold *R*²	95% bootstrap CI	RMSE	MAE	Spearman *ρ*
DynaTab	0.626	[0.539, 0.727]	2.99	1.76	0.888
**Stacking ensemble**	**0.613**	**[0.510, 0.726]**	**3.04**	**1.82**	**0.901**
Random forest	0.609	[0.501, 0.736]	3.06	1.81	0.896
Bagging	0.599	[0.485, 0.721]	3.10	1.84	0.896
KNN	0.580	[0.503, 0.672]	3.17	2.01	0.884
Lasso	0.579	[0.484, 0.690]	3.17	2.01	0.900
SVR (RBF)	0.578	[0.485, 0.689]	3.18	1.96	0.857
Extra trees	0.575	[0.470, 0.697]	3.19	1.85	0.904
ElasticNet	0.571	[0.490, 0.669]	3.20	2.10	0.900
Ridge	0.562	[0.477, 0.656]	3.23	2.19	0.885
Bayesian ridge	0.561	[0.476, 0.657]	3.24	2.20	0.887
MLP	0.522	[0.409, 0.612]	3.38	2.31	0.812
Gradient boosting	0.448	[0.232, 0.624]	3.63	2.11	0.873
Linear regression	0.312	[–0.146, 0.564]	4.05	2.58	0.794

All preprocessing (multiple imputation, standardization, four-method consensus feature selection, and hyperparameter tuning) was performed inside the training partition of each outer fold. Metrics are computed once on the pooled out-of-fold prediction vector (averaged across five repetitions) rather than averaged over folds. The 95% confidence intervals are percentile intervals from 2,000 bootstrap resamples of countries. All 14 evaluated models are listed (the 13 conventional models and the high-dimensional DynaTab benchmark); the best-performing conventional model is shown in bold. Sensitivity to the fold partition is reported in **Supplementary Table S8**, and results without any feature-selection step are presented in **Supplementary Table S9**. DynaTab is a high-dimensional deep tabular model evaluated under the identical protocol with seed ensembling to control the initialization variance of a neural model at this sample size; its pooled *R*² (0.626) is statistically comparable to the stacking ensemble.

RMSE, root mean square error; MAE, mean absolute error; *ρ*, Spearman rank correlation between predicted and observed rates.

### 2.3 Data preprocessing

#### 2.3.1 Country harmonization and inclusion criteria

The countries included in this analysis were harmonized using the United Nations M49 numeric codes. For HDI and WHO obesity data for which only country names were available, automated fuzzy string matching (Levenshtein distance threshold ≥ 60%) was applied as an initial step. All automated matches were subsequently verified manually by two investigators against ISO 3166–1 country name lists and UN M49 designations. Matches with similarity between 60% and 75% were subject to additional cross-referencing against country codes and geographic context; ambiguous matches (*n* = 14) were resolved through consensus. No automated match was accepted without manual confirmation. Following harmonization, a total of 163 countries had complete data from all four primary sources (88% of GLOBOCAN countries were represented). No formal sample size calculation was performed, as the study comprised all countries with available data. Inclusion required non-missing GLOBOCAN 2022 incidence ASR, FAOSTAT food supply availability data for 2015–2019, and HDI data for 2023. A total of 22 countries could not be matched, which are primarily small island states not individually reported in the FAOSTAT food balance sheets.

#### 2.3.2 Missing data strategy

Missing feature values were imputed using Multiple Imputation by Chained Equations (MICE) via the scikit-learn IterativeImputer (34), with each feature predicted from all others via Bayesian Ridge Regression (10 convergence iterations). Features with more than 35% missingness were excluded prior to imputation, retaining only features with at least 65% completeness. MICE was preferred over simple median imputation because it preserves the covariance structure of the data and produces less biased estimates under the missing at random (MAR) assumption (35). In the final analytical file, the dietary variables are complete, and the only missing values are the HDI-derived columns for the two countries absent from the UNDP annex (French Polynesia and New Caledonia), 16 cells in total or 0.07% of the feature matrix. Imputation is nevertheless performed inside each training fold rather than once on the whole dataset, with the number of donor features limited to the 20 most strongly related predictors for computational tractability. Sensitivity analyses reporting mean, SD, and Spearman correlation changes before and after imputation for all imputed variables are provided in **Supplementary Table S4**.

#### 2.3.3 Normalization

All numerical features were standardized to zero mean and unit variance (*z*-score normalization) using the StandardScaler function from the scikit-learn library, implemented within a sklearn Pipeline object. Critically, the StandardScaler was fit exclusively on the training portion of each cross-validation fold and applied (without refitting) to the held-out validation fold, ensuring that no information from validation data influenced feature scaling and precluding data leakage from the normalization step. Standardization is particularly important for distance-based and gradient-based algorithms (SVR, KNN, and MLP) and does not alter tree-based model performance; all models were evaluated within the same pipeline for consistency.

### 2.4 Feature engineering

A total of 155 features were engineered from the raw data, organized into six methodological categories. Feature engineering improves model performance and enables capture of complex dietary relationships beyond individual food items (18).

#### 2.4.1 Composite dietary indices

Four composite dietary indices were computed. The first is the iodine-rich food availability index. It is named an availability index because it measures national food-group availability and not iodine intake or iodine exposure. It combines the per-capita food supply availability (kg/capita/year) of the three food groups identified by WHO/FAO and by Zimmermann and Boelaert (36) as the principal dietary sources of iodine in populations worldwide—seafood (the sum of seven FAOSTAT categories: freshwater fish, demersal fish, pelagic fish, other marine fish, crustaceans, cephalopods, and mollusks), dairy (milk excluding butter), and eggs as a kilogram-equivalent weighted sum: index = seafood + 0.10 × dairy + 0.05 × eggs. The weights are not equal, and they are not derived from measured iodine concentrations, which FAOSTAT does not report. They are order-of-magnitude scaling constants chosen *a priori* so that the three groups enter on a comparable per-capita mass scale: national dairy supply is roughly an order of magnitude larger in kg/capita/year than seafood supply, and egg supply is intermediate, so an unweighted sum of raw supply quantities would be numerically dominated by dairy tonnage rather than by iodine-relevant food availability. Under this construction, seafood accounts for 90.1% of the variance of the index, dairy for 3.3%, and eggs for 0.1%. Because any such weighting is an analytical choice rather than a measurement and because the index may also be read as a marker of animal-origin food availability more generally, its construction is examined in a dedicated set of sensitivity analyses described in Section 2.7.2 and reported in Section 3.5. The remaining three indices were defined as follows. The Mediterranean Diet Score Proxy (MDS) was computed as the sum of *z*-scores for beneficial components (fruits, vegetables, olive oil, legumes, cereals, nuts, and fish) minus *z*-scores for detrimental components (red meat, sugar, and animal fats), following the framework of Trichopoulou et al. (37) adapted for country-level food supply data. The Western Diet Score Proxy (WDS) summed *z*-scores for red meat, processed meat, sugar, sweeteners, animal fats, vegetable oils, and cream. The Dietary Diversity Index (DDI) counted the number of food items with supply exceeding 1 kg/capita/year per country, reflecting dietary variety (28).

#### 2.4.2 Nutrient density and ratio measures

Nutrient density metrics were computed to capture dietary quality independent of total energy intake: animal protein density (g per 1,000 kcal), plant protein density, total fat density, and the animal-to-plant protein ratio. The seafood protein fraction (seafood (kg) divided by total animal product (kg)) was calculated to measure the relative contribution of marine protein to the national diet.

#### 2.4.3 Ultra-processed food proxy

In the absence of individual-level NOVA classification data at the country level, we constructed an exploratory ultra-processed food (UPF) proxy from FAOSTAT items partially overlapping with UPF categories. This proxy captures only a subset of ultra-processed products and should be interpreted as an approximation: sugar (raw equivalent), sweeteners, beer, wine, and alcoholic beverages. Both absolute food supply availability (kg/capita/year) and the energy fraction (UPF kcal/total kcal) were computed. While this proxy does not capture the full spectrum of ultra-processed products, it serves as a food-supply-level approximation consistent with the approach used by Monteiro et al. (38).

#### 2.4.4 Interaction terms and temporal lags

Five interaction terms were created between key dietary variables and HDI (seafood × HDI, dairy × HDI, iodine proxy × HDI, animal protein × HDI, and Mediterranean score × HDI) to test for effect modification, whether dietary associations differ by development level. For the lag sensitivity analysis, seafood supply averaged over windows ending 0, 3, 5, 10, and 12 years before the outcome year was correlated with the 2022 cancer outcome, generating a lag profile spanning 0–12 years of dietary exposure. These windows overlap and are highly autocorrelated, so they are treated as a sensitivity check rather than as independent tests.

### 2.5 Feature selection

From the 150 numeric predictors, a four-method consensus ranking was used to retain the 60 highest-ranked variables for modeling: (1) mutual information regression, capturing nonlinear dependence between each feature and the outcome, (2) absolute Spearman rank correlation, (3) univariate *F*-test scores, and (4) random forest impurity-based importance. Features were ordered by their mean rank across the four criteria. The entire selection procedure together with imputation, standardization, and hyperparameter search is refitted from scratch within the training partition of every outer cross-validation fold, so the countries used to evaluate a model never contribute to choosing its features. Because the selected subset varies from fold to fold, we additionally report which features were chosen most frequently across folds (**Supplementary Table S7**) and repeat the main models on the complete 150-variable matrix with no selection step at all (**Supplementary Table S9**). The multi-method consensus reduces the selection bias inherent in any single ranking criterion (39), while nesting the procedure inside cross-validation removes the optimistic bias that arises when selection precedes validation (40, 41). A comprehensive inventory of all 155 variables in the analytical file, including source, reference year, unit, normalization, missingness proportion, imputation status, and lag status, is provided as **Supplementary Table S5**.

### 2.6 Machine learning modeling

#### 2.6.1 Regression task

A total of 13 regression models were evaluated: unregularized linear regression, ridge, Lasso, ElasticNet, Bayesian Ridge, K-nearest neighbors, support vector regression (RBF kernel), random forest (300 trees), Extra Trees (300 trees), gradient boosting (300 trees, learning rate 0.03), bagging (100 estimators), a multi-layer perceptron (64–32 hidden units, ReLU, Adam, early stopping), and a stacking ensemble (random forest, gradient boosting, ElasticNet, and KNN as base learners, with Ridge as meta-learner). Every model was embedded in a scikit-learn Pipeline and assessed by nested cross-validation. The outer loop consisted of 10 shuffled folds repeated five times (50 outer fits per model); within each outer training partition, an inner fivefold shuffled cross-validation was used for hyperparameter search (KNN: k ∈ {3, 5, 7, 9, 11}; SVR: C ∈ {0.1, 1, 5, 10, 50}; Ridge: α ∈ {0.1, 1, 10, 100}; Lasso: α ∈ {0.001, 0.01, 0.1, 1}; ElasticNet: α ∈ {0.01, 0.1, 1} × l1-ratio ∈ {0.2, 0.5, 0.8}) and for the internal folds of the stacking meta-learner. Because the analytical file is ordered by outcome magnitude, all inner and outer splits were shuffled under a fixed seed; unshuffled splitting was found to distort the stacking meta-learner and is explicitly avoided. Rather than averaging fold-level *R*² values, which are not independent and, with roughly 16 countries per held-out fold, are highly unstable, we pooled the out-of-fold prediction of every country into a single prediction vector (averaged over the five repetitions) and computed *R*², RMSE, MAE, and Spearman *ρ* once on that vector. Confidence intervals were obtained by resampling countries with replacement (2,000 bootstrap replicates), which propagates the sampling variability of the 163 units of analysis rather than the variability of arbitrary fold boundaries. Sensitivity to the fold partition is reported separately as the standard deviation of *R*² across the five repetitions (**Supplementary Table S8**).

#### 2.6.2 Classification task

A binary classification task was also examined. It is prespecified and reported as a sensitivity analysis only since dichotomizing a continuous rate discards information, and the median of the incidence distribution carries no clinical meaning. Countries were split at the median age-standardized rate (3.7 per 100,000; *n* = 82 at or above, *n* = 81 below) and, as a check on threshold dependence, at the upper quartile of the distribution. Seven classifiers were evaluated (logistic regression with L2 penalty and C = 0.5, KNN, SVM with RBF kernel, random forest, Extra Trees, gradient boosting, and an MLP neural network) using the same nested design as the regression task, with stratified outer folds. Discrimination (AUC-ROC, AUPRC), calibration (Brier score, calibration curves), and threshold metrics (accuracy, F1) were computed from pooled out-of-fold predicted probabilities with country-level bootstrap confidence intervals. Full results are given in **Supplementary Table S12** and **Supplementary Figures S8** and **S9**, and no conclusion in this manuscript rests on them.

#### 2.6.3 Block-wise models and the incremental value of dietary features

Because national food supply composition is itself strongly patterned by development level, a model containing both dietary and socioeconomic variables cannot show how much of its accuracy is genuinely dietary. We therefore fitted three parallel model families under the identical nested cross-validation protocol: a socioeconomic-only block (HDI, its life expectancy, education and income sub-indices, obesity prevalence, and the four standardized, quadratic, and interaction terms derived from them; nine variables in total, the categorical HDI class being excluded), a diet-only block (all FAOSTAT supply, nutrient-density, and composite dietary variables; 140 variables), and the combined set. The incremental value of dietary information was quantified as the difference in pooled out-of-fold *R*² between the combined and socioeconomic-only models, with a paired country-level bootstrap (4,000 replicates) providing a confidence interval and a two-sided bootstrap *p*-value for that difference.

#### 2.6.4 Region-held-out validation

Neighboring countries resemble one another in diet, registry infrastructure, and screening practice, so random cross-validation folds are not independent: a country held out at random is usually accompanied in the training set by several of its regional peers. To test whether the models generalize beyond that similarity, we repeated the analysis with entire world regions held out. Countries were assigned to six regions (Africa; Asia; Europe; Northern America and the Caribbean; Oceania; and South America) from their ISO-3166 codes, and each region was predicted, in turn, by a model trained only on the remaining regions, with feature selection and tuning again refitted inside each training set. Performance is reported per held-out region and pooled across regions, and we report rank agreement alongside *R*² because the two can diverge sharply when a model ranks countries correctly but predicts the wrong level. Region was additionally included as a set of fixed-effect indicators in the extended confounder-adjustment model described in Section 2.7.1.

### 2.7 Statistical association analyses

#### 2.7.1 Bivariate and adjusted correlations

A total of 12 dietary and metabolic variables were related to thyroid cancer incidence using Spearman rank correlations. Three association models are reported for each variable: an unadjusted correlation, a partial correlation adjusted for HDI and obesity prevalence, and an extended partial correlation additionally adjusted for the three HDI sub-indices (life expectancy, education, and income) and for region indicators, which together act as proxies for health-system capacity, registry development, and geographic clustering. Partial correlations were computed on the residuals of the dietary variable and of incidence after regression on the confounder set, using the 161 countries with complete socioeconomic data. Because 12 variables were examined under three models, *p*-values within each family were corrected both by the Benjamini–Hochberg false discovery rate procedure (42) and, more conservatively, by Bonferroni adjustment; corrections were applied within each of the three families, including the two adjusted families, so that the evidential status of an adjusted estimate is not overstated. FDR-adjusted values are reported in **Table 2** and both corrections, for all three families, in **Supplementary Table S6**. Adjusted associations that survive correction are described as robust ecological associations; those that do not are described as exploratory, and no dietary conclusion is drawn from an uncorrected *p*-value.

**Table 2:** Unadjusted and confounder-adjusted associations between dietary factors and thyroid cancer incidence ASR, with false discovery rate correction across all 12 comparisons in each family (*n* = 161 countries with complete socioeconomic data).

Dietary factor	Unadjusted *ρ*	*q* (FDR)	Partial *r* (HDI + obesity)	*q* (FDR)	Partial *r* (extended set)	*q* (FDR)
Iodine-rich food availability index	0.234	0.016*	0.231	0.019*	0.224	0.026*
Total energy (kcal/capita/day)	0.230	0.016*	0.098	0.309 ns	0.028	0.794 ns
Animal/plant protein ratio	0.221	0.016*	0.246	0.019*	0.260	0.011*
Seafood supply (kg/capita/year)	0.219	0.016*	0.208	0.032*	0.196	0.031*
Animal protein (g/capita/day)	0.190	0.035*	0.177	0.059 ns	0.176	0.051 ns
Mollusks (kg/capita/year)	0.187	0.035*	0.184	0.059 ns	0.203	0.031*
Western diet score	0.181	0.037*	0.043	0.710 ns	0.040	0.739 ns
Dairy supply (kg/capita/year)	0.176	0.038*	0.158	0.089 ns	0.198	0.031*
Obesity prevalence (%)	0.170	0.042*	0.020	0.872 ns	0.157	0.079 ns
Mediterranean diet score	0.141	0.080 ns	0.145	0.113 ns	0.127	0.145 ns
Total fat density	0.066	0.406 ns	0.007	0.925 ns	0.008	0.918 ns
Plant protein (g/capita/day)	–0.167	0.042*	–0.095	0.309 ns	–0.149	0.088 ns

The basic adjustment set contains HDI and obesity prevalence; the extended set adds the three HDI sub-indices (life expectancy, education, and income) and region fixed effects. The Bonferroni-adjusted values for all three families, including the extended adjustment set, are given in **Supplementary Table S6**; under Bonferroni correction, two associations remain significant in the basic-adjustment family—the animal-to-plant protein ratio (*p* = 0.019) and the iodine-rich food availability index (*p* = 0.038)—and one in the extended family—the animal-to-plant protein ratio (*p* = 0.011), with the iodine-rich food availability index borderline (*p* = 0.052). The iodine-rich food availability index is the composite defined in Section 2.4.1; sensitivity analyses of its construction are reported in **Table 3**. *ρ*, Spearman rank correlation; Partial *r*, Pearson partial correlation of residuals; ns, not significant at *q* < 0.05; *q*, Benjamini–Hochberg adjusted *p*-value computed within each family of 12 comparisons. *q < 0.05; **q < 0.01; ***q < 0.001.

**Table 3:** Sensitivity analyses for the iodine-rich food availability index: individual components, alternative weighting schemes, and specificity against iodine-poor animal-origin foods (*n* = 161 countries with complete socioeconomic data).

Variable	Unadjusted *ρ*	Partial *r*, basic (*q*)	Partial *r*, extended (*q*)	Partial *r*, extended + animal protein (*q*)
Panel A: Individual components entered separately				
Seafood supply (kg/capita/year)	0.219	0.208 (0.016)	0.196 (0.017)	0.176 (0.034)
Dairy supply, milk excluding butter (kg/capita/year)	0.176	0.158 (0.054)	0.198 (0.017)	0.190 (0.032)
Egg supply (kg/capita/year)	0.217	0.152 (0.054)	0.065 (0.411)	0.024 (0.762)
Iodine-rich food availability index (primary)	0.234	0.232 (0.013)	0.224 (0.017)	0.203 (0.032)
Panel B: Alternative weighting schemes				
Primary index (1.00/0.10/0.05)	0.234	0.232 (0.004)	0.224 (0.006)	0.203 (0.012)
Equal-weighted sum (1/1/1)	0.279	0.246 (0.003)	0.250 (0.004)	0.229 (0.009)
Equal-weighted sum of *z*-scores	0.293	0.270 (0.002)	0.238 (0.004)	0.206 (0.012)
Iodine-content-weighted (1.00/0.50/0.83)	0.291	0.267 (0.002)	0.256 (0.004)	0.231 (0.009)
Seafood only (1/0/0)	0.219	0.208 (0.008)	0.196 (0.013)	0.176 (0.025)
Panel C: Specificity against animal-origin food availability				
Iodine-rich food availability index	0.234	0.232 (0.013)	0.224 (0.018)	0.203 (0.039)
Iodine-poor animal-origin food index (bovine, pig, poultry, mutton, and goat meat)	0.309	0.135 (0.087)	0.102 (0.198)	0.088 (0.537)
Total animal protein supply (g/capita/day)	0.190	0.177 (0.033)	0.176 (0.035)	—
Total animal products supply (kg/capita/year)	0.225	0.190 (0.032)	0.175 (0.035)	0.024 (0.961)

Values are the partial correlation with thyroid cancer incidence ASR with the Benjamini–Hochberg adjusted *q*-value in parentheses; corrections are applied within each panel, so the same variable may carry slightly different *q*-values in different panels. Basic adjustment refers to HDI and obesity prevalence; extended adjustment refers to basic plus the three HDI sub-indices and region fixed effects; the final column adds total animal protein supply to the extended set. The primary index is seafood + 0.10 × dairy + 0.05 × eggs (Section 2.4.1). Iodine-content weights are ratios of approximate relative iodine concentrations assumed a priori for the three food groups (about 300, 150, and 250 μg/kg fresh weight). The iodine-poor animal-origin food index is the sum of bovine, pig, poultry, and mutton and goat meat supply. Dashes mark estimates that are not interpretable because the variable is itself contained in the adjustment set. Full output, including the joint multivariable model of the three components and variance inflation factors, is given in **Supplementary Tables S13**–**S16** and **Supplementary Figure S16**.

#### 2.7.2 Sensitivity analyses for the iodine-rich food availability index

The iodine-rich food availability index is a composite of three food groups combined with analyst-chosen weights, and it measures availability rather than intake. Four prespecified sensitivity analyses were therefore conducted to establish what the index actually captures, all of them using the same two adjustment sets, the same 161 countries, and the same Benjamini–Hochberg procedure as in Section 2.7.1, with the correction applied within each new family of comparisons. First, the three components—seafood supply, dairy supply (milk excluding butter), and egg supply—were entered as separate exposures in place of the composite to determine whether the association is consistently distributed across iodine-related food groups or driven by a single component. Second, the three components were entered simultaneously as standardized terms in a single multivariable model under both adjustment sets, with variance inflation factors reported, so that their contributions could be assessed net of one another. Third, the composite was rebuilt under three alternative weighting schemes and compared with the primary construction and with a seafood-only variant: an equal-weighted sum of raw supply quantities (1/1/1), an equal-weighted sum of the three standardized components, an iodine-content-weighted sum in which the weights are ratios of approximate relative iodine concentrations assumed a priori for the three food groups (about 300, 150, and 250 µg/kg fresh weight for seafood, dairy, and eggs, respectively), giving weights of 1.00/0.50/0.83. Fourth, to test whether the index reflects a broader pattern of animal-origin food availability rather than iodine-rich food availability specifically, we (a) added total animal protein supply to the extended adjustment set and re-estimated every association and (b) constructed an iodine-poor animal-origin food index from the four FAOSTAT meat categories that are poor dietary sources of iodine (bovine meat, pig meat, poultry meat, and mutton and goat meat) and tested it as a negative control alongside total animal protein supply and total animal products supply. If the association reflects animal-origin food availability in general, the control index should behave like the iodine index under adjustment; if it reflects iodine-rich food groups specifically, it should not.

#### 2.7.3 Temporal lag panel analysis

To assess the long-term effect of nutrition on cancer, lagged food supply variables retained in the analytical dataset (0, 3, 5, 10, and 12 years before the outcome year) were each correlated with 2022 incidence using Spearman rank correlation. This generates a lag profile spanning 0–12 years, enabling the evaluation of whether the diet–cancer correlation strengthened over time—indicative of true long-term dietary effects—or remained relatively stable, suggesting potential confounding by shared characteristics across countries.

#### 2.7.4 Descriptive path decomposition

To quantify the degree to which HDI accounts for the ecological association between seafood food supply availability and thyroid cancer incidence, a path-analytic decomposition was conducted following the framework of Baron and Kenny (43). This approach is applied here in a descriptive, path-analytic sense to partition the ecological correlation into HDI-correlated and HDI-independent components. It does not establish causal mediation, as the cross-sectional ecological design precludes the temporal and counterfactual requirements of formal causal mediation analysis; accordingly, no causal interpretation is attached to any path. Four path coefficients were estimated using OLS regression: path a (seafood → HDI), path b (HDI → cancer | seafood), the total seafood–incidence coefficient c, and the seafood–incidence coefficient conditional on HDI (c′). The indirect component was computed as a × b and the share attributable to the HDI pathway as the ratio of that component to the total association. Because this share is a ratio of regression coefficients, it is unstable in samples of this size, so we bootstrapped it over countries (5,000 replicates) and report the resulting interval alongside the point estimate.

#### 2.7.5 Secondary analysis: mortality and differential cancer detection

Thyroid cancer mortality was prespecified as a secondary outcome with a single interpretive purpose. Because thyroid cancer is highly survivable and its incidence is strongly influenced by diagnostic intensity, while its mortality is comparatively insensitive to the detection of small, indolent, screen-detected tumors, a comparison of the two outcomes provides an indirect check on whether the incidence associations reported in Sections 3.2–3.6 are attributable to differential detection. We therefore related the same eight dietary and socioeconomic predictors to three outcomes—incidence ASR, mortality ASR, and the mortality-to-incidence ratio—using Spearman rank correlations across the 163 countries. The reasoning is asymmetric and is stated here to avoid over-interpretation: associations that are present for incidence but absent for the mortality-to-incidence ratio are consistent with a detection-driven pattern, whereas the converse would argue against one. No dietary conclusion of this study is drawn from the mortality analysis, and mortality is not modeled, decomposed, or used in any predictive comparison.

### 2.8 Explainability and interpretability

Four complementary interpretability methods were applied: (1) Permutation importance was computed exclusively on the held-out validation fold within each cross-validation iteration (30 permutation repeats per fold) and then averaged across folds to yield mean Δ*R*² ± SD for gradient boosting (GBM) and random forest (RF). Computing importance on held-out data rather than training data prevents overestimation of feature relevance. (2) Permutation-based feature contribution analysis was computed by replacing each feature value for each country with values drawn from a background distribution of 50 randomly sampled country profiles, taking the difference in model prediction as the contribution estimate and averaging across the 50 background samples. This was applied to both GBM and RF models independently. (3) Partial dependence plots (PDPs) were computed for the top eight features using both GBM and RF, displaying the marginal effect of each feature on predicted incidence averaged over all other feature values. (4) Dietary pattern analysis used PCA to identify latent dietary patterns and K-means clustering (optimal *k* determined by silhouette score) to identify dietary cluster profiles; Kruskal–Wallis tests compared thyroid cancer incidence across clusters. All four methods are used descriptively; because the feature matrix is extensively collinear, none of them identifies a unique set of influential variables, and this is stated wherever the results are reported.

### 2.9 Software and reproducibility

Analyses were run in Python 3 with scikit-learn (44), pandas, NumPy, SciPy, Matplotlib, and Seaborn; figures were produced at 300 dpi. The complete analysis pipeline is released rather than described: **Supplementary Code S1** is a single executable script that reads the analytical dataset and regenerates every number, table, and figure reported here, the nested cross-validation loops, the block-wise and region-held-out analyses, the association models with both multiplicity corrections, the sensitivity analyses of the iodine-rich food availability index, the path decomposition, and all bootstrap intervals under a fixed random seed (seed = 42). The script also contains the code that rebuilds the HDI sub-indices from the UNDP annex so that the socioeconomic reconstruction described in Section 2.2.3 can be verified independently. The harmonized country-level dataset is provided as **Supplementary Data S1** in CSV and XLSX form with a variable dictionary; together with **Supplementary Code S1**, these files allow the analysis to be re-executed end to end without contacting the authors.

## 3 Results

### 3.1 Study population and descriptive statistics

The analytical dataset comprised 163 countries harmonized on UN M49 codes with 155 variables (113 FAOSTAT food supply variables across four nutritional domains, six socioeconomic variables, 32 derived or composite features, and four outcome-related variables), as itemized in the data dictionary accompanying **Supplementary Data S1**. Five of these variables are outcome-related or categorical and are excluded from the feature matrix to avoid circularity—the incidence ASR itself, the mortality ASR, the log-transformed incidence, the median-split burden indicator, and the categorical HDI class—leaving 150 numeric predictors. Age-standardized thyroid cancer incidence, the primary outcome, ranged from 0.5 to 31.8 per 100,000, with a median of 3.7 (IQR 1.2–7.7) and a mean of 5.11 (SD 4.90). The distribution was strongly right-skewed (Shapiro–Wilk W = 0.83, *p* = 1.20 × 10^−12^), which is why rank-based statistics are used for all association analyses, and it was log-transformed before any parametric analysis (Shapiro–Wilk *W* = 0.95, p = 1.27 × 10^−5^ after transformation; **Supplementary Figure S1**). Mortality rates were an order of magnitude lower than incidence rates, consistent with the generally favorable prognosis of thyroid cancer.

**Figure 2** shows the 30 countries with the highest incidence rates. The three highest were Cyprus (ASR = 31.8), the Republic of Korea (ASR = 23.2), and French Polynesia (ASR = 16.9). Panel B shows the 30 countries with the highest mortality-to-incidence ratios, which are predominantly lower-HDI countries; that contrast is examined in the secondary analysis in Section 3.7. The distribution of all outcome variables, including the log transformation and the incidence–mortality relationship by HDI, is given in **Supplementary Figure S1**.

**Figure 2:**
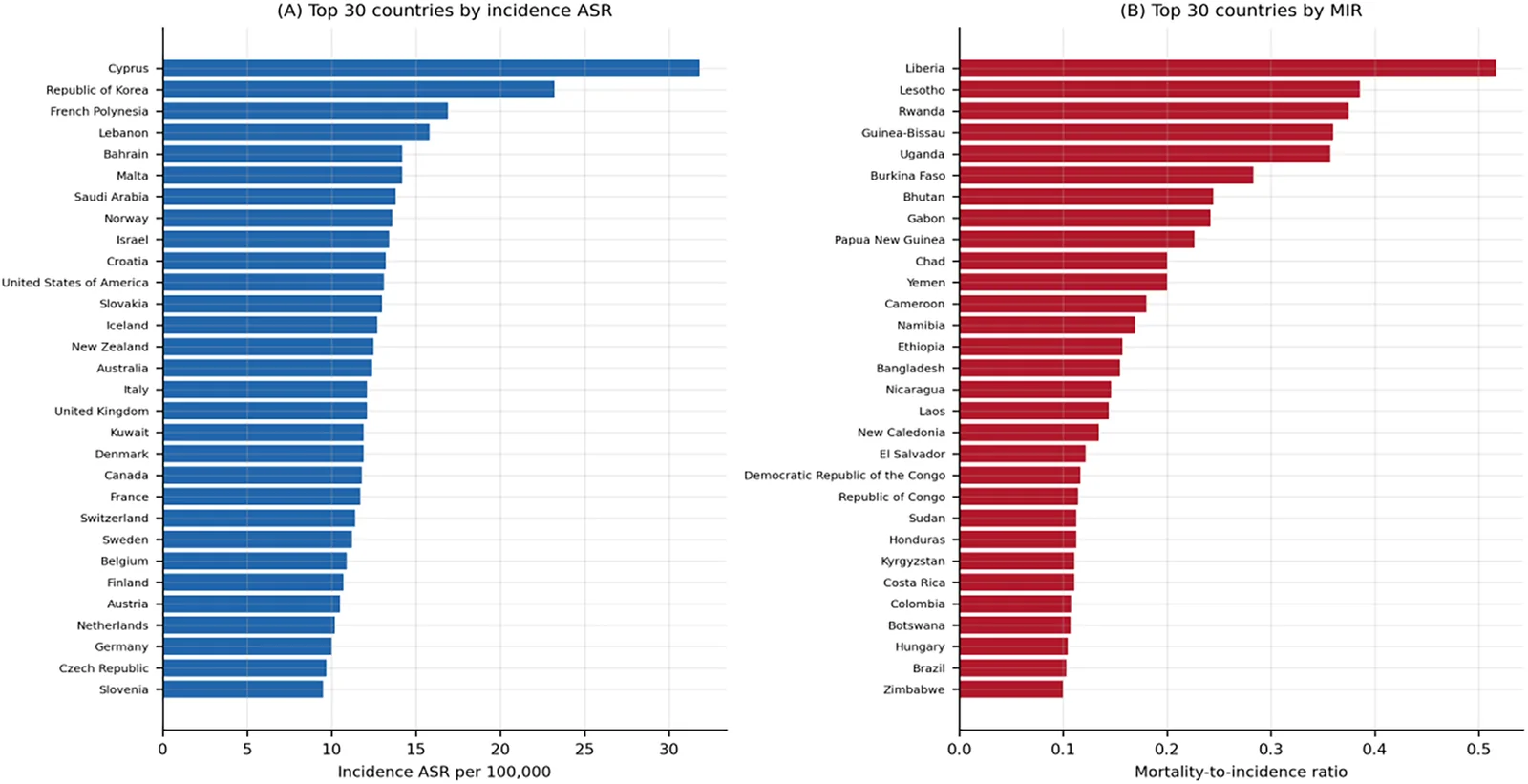
Global thyroid cancer burden in 2022. **(A)** The 30 countries with the highest age-standardized incidence rates per 100,000 (world standard population), led by Cyprus (31.8), the Republic of Korea (23.2), and French Polynesia (16.9). **(B)** The 30 countries with the highest mortality-to-incidence ratios, which are predominantly lower-HDI countries and stand in contrast to **(A)**; this contrast is examined in the secondary analysis in Section 3.7.

### 3.2 Prediction of national thyroid cancer incidence

The first objective was to establish how much of the between-country variation in thyroid cancer incidence national dietary and socioeconomic data can explain. **Table 1** and **Figure 3** present the comparison of the 13 regression algorithms under the nested design. Stacking ensemble performed best, with a pooled out-of-fold *R*² of 0.613 (95% country-level bootstrap CI [0.510, 0.726]), RMSE = 3.04, MAE = 1.82, and Spearman *ρ* = 0.901 between predicted and observed rates. Random forest (*R*² = 0.609), bagging (*R*² = 0.599), and KNN (*R*² = 0.580) followed. Three observations matter more than the ranking. First, these estimates are leakage-free: because the consensus ranking, the imputation, the scaling, and the hyperparameter search are all refitted inside each training fold, no held-out country contributed to the model that predicted it. Second, the advantage of ensembles over regularized linear models is real but small (0.613 versus 0.579 for Lasso), so our first prespecified expectation is only marginally supported; unregularized linear regression, by contrast, performed clearly worse (*R*² = 0.312) and remains the appropriate cautionary comparator. Third, rank agreement was consistently higher than *R*² (*ρ* ≈ 0.901 for the leading models), meaning that the models order countries better than they reproduce their absolute rates: all four leading models recover the broad gradient but compress it, and the highest-incidence countries, Cyprus and the Republic of Korea, in particular, are consistently underpredicted (**Supplementary Figure S7**). Since both are documented examples of screening-driven overdiagnosis, this systematic shortfall is better explained by unmeasured detection intensity than by dietary composition. Repeating the four models on the complete 150-variable matrix with no selection step gave comparable performance (**Supplementary Table S9**; for example, random forest *R*² = 0.622 versus 0.609 with nested selection), indicating that the consensus ranking neither creates nor destroys predictive signal once it is nested, and sensitivity to the fold partition was small (**Supplementary Table S8**).

**Figure 3:**
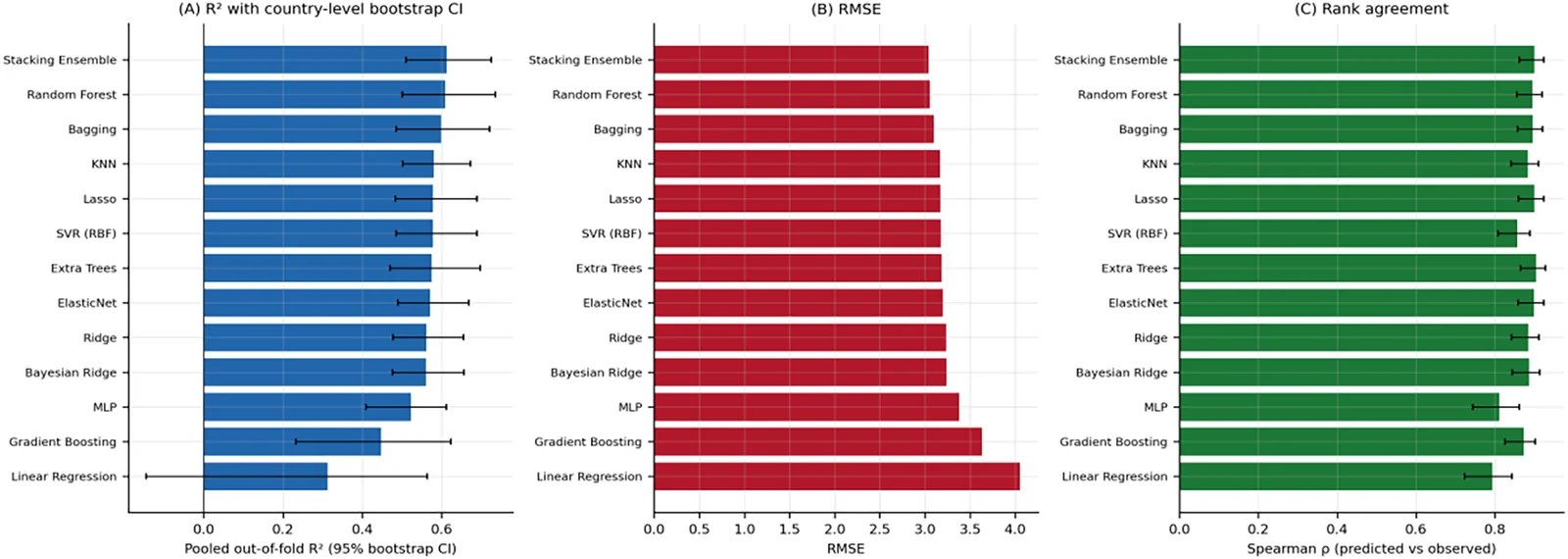
Regression model comparison under nested cross-validation, with thyroid cancer incidence ASR as the outcome. **(A)** Pooled out-of-fold *R*² with 95% country-level bootstrap confidence intervals. **(B)** RMSE. **(C)** Spearman *ρ* between predicted and observed rates with bootstrap intervals. Models are sorted by *R*². All estimates derive from predictions for countries absent from every step of model construction, including imputation and feature selection. The high-dimensional DynaTab benchmark is reported in **Table 1** and Section 4.2.1 and is not shown in this panel of conventional models.

Because neighboring countries resemble one another in diet, registry infrastructure, and screening practice, we also repeated the analysis with entire world regions held out (**Supplementary Table S11**; **Supplementary Figure S15**). Pooled across the six held-out regions, *R*² fell to 0.558 for random forest and 0.453 for Bayesian Ridge, compared with 0.609 and 0.561 under random cross-validation, while rank agreement was largely preserved (pooled *ρ* = 0.813 and 0.850). Region-specific *R*² was negative in three of the six regions for random forest and extremely so for South America (*R*² = –95.8, *n* = 10), where a narrow within-region range of incidence makes the variance denominator very small; the best-predicted region was Northern America and the Caribbean (*R*² = 0.598). The models therefore transport the ordering of countries across regions reasonably well but not their level, and part of the apparent accuracy under random cross-validation comes from geographic similarity between training and test countries. We accordingly describe the models as descriptive summaries of the present sample rather than as prediction tools for unobserved regions.

### 3.3 Incremental value of dietary information over development indicators

The second objective was to determine whether the accuracy reported above is dietary or socioeconomic. **Figure 4** and **Supplementary Table S10** report the block-wise comparison, and it is the most important negative result in this manuscript. A socioeconomic-only model containing nine variables achieved a pooled out-of-fold *R*² of 0.609 with Bayesian Ridge, 0.589 with random forest, and 0.558 with Extra Trees. A diet-only model built from 140 dietary variables achieved far less at 0.284, 0.257, and 0.228 respectively. The combined model reached 0.561, 0.609, and 0.575. The incremental contribution of the entire dietary feature set over the nine socioeconomic indicators was therefore Δ*R*² = −0.048 (95% CI −0.169 to +0.034, bootstrap *p* = 0.266) for Bayesian Ridge, +0.021 (−0.059 to +0.079, *p* = 0.579) for random forest, and +0.017 (−0.084 to +0.133, *p* = 0.754) for Extra Trees. None of the three intervals excludes zero, and for Bayesian Ridge the point estimate is negative. Adding 140 dietary variables to nine development indicators therefore does not improve the prediction of national thyroid cancer incidence by any margin these data can distinguish from zero. We state this as an absence of demonstrable gain rather than as proof of no gain: the upper bounds of the intervals reach +0.079 and +0.133, so a modest dietary contribution cannot be excluded at this sample size. This is why we describe the predictive performance reported in Section 3.2 as socioeconomic rather than dietary and why the dietary findings of this study rest on the confounder-adjusted association analysis rather than on model accuracy.

**Figure 4:**
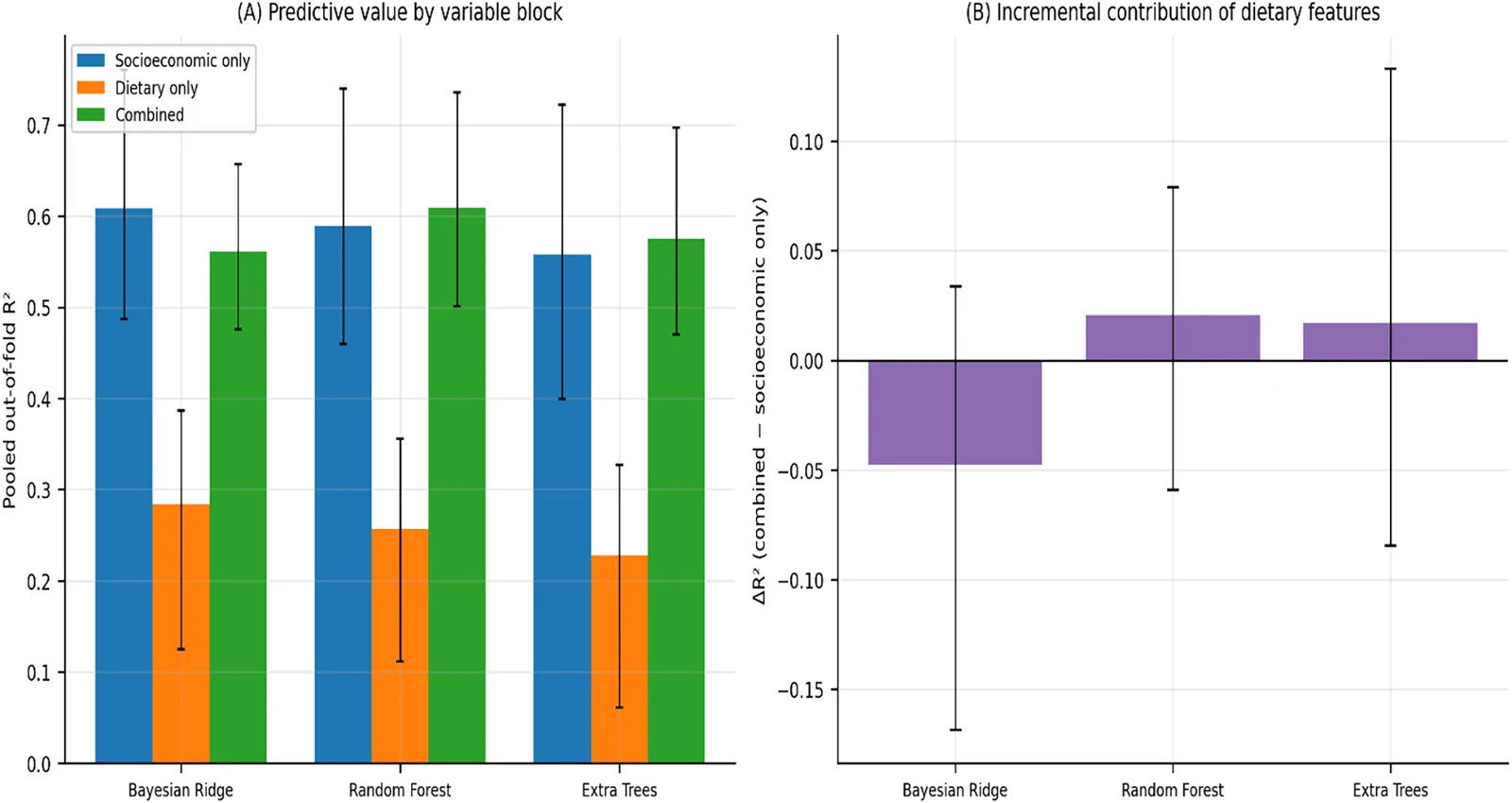
Incremental predictive value of dietary features. **(A)** Pooled out-of-fold *R*² for socioeconomic-only, diet-only, and combined feature blocks under identical nested cross-validation, with 95% country-level bootstrap intervals. **(B)** Δ*R*² for the combined model relative to the socioeconomic-only model, with paired bootstrap intervals. Nine development variables outperform 140 dietary variables, and no incremental interval excludes zero.

Held-out permutation importance reaches the same conclusion by a different route (**Figure 5**). The ranking is dominated by socioeconomic rather than dietary variables in both models: the three highest-ranked features were income index, life expectancy index and HDI squared for random forest, and income index, sugar cane, and life expectancy index for Gradient Boosting, with the income sub-index alone accounting for a mean Δ*R*² of 0.273 in the random forest model, an order of magnitude larger than any dietary feature. Two cautions apply. Permutation importance distributes credit arbitrarily among correlated predictors, and this feature matrix is extensively collinear, so the appearance of one food group rather than a closely related one carries little information, and high importance in a model describes how the model uses a variable, not how strongly that variable relates to disease risk. Feature ranking stability across the six principal models is reported in **Supplementary Table S7**. Partial dependence curves for the eight highest-ranked features, a permutation-based decomposition of individual country predictions, and the same contributions stratified by development level are provided as **Supplementary Figures S10**–**S12**; all three are model summaries computed in a strongly collinear feature space and are not read as dose–response relationships or as evidence of diet–development interaction.

**Figure 5:**
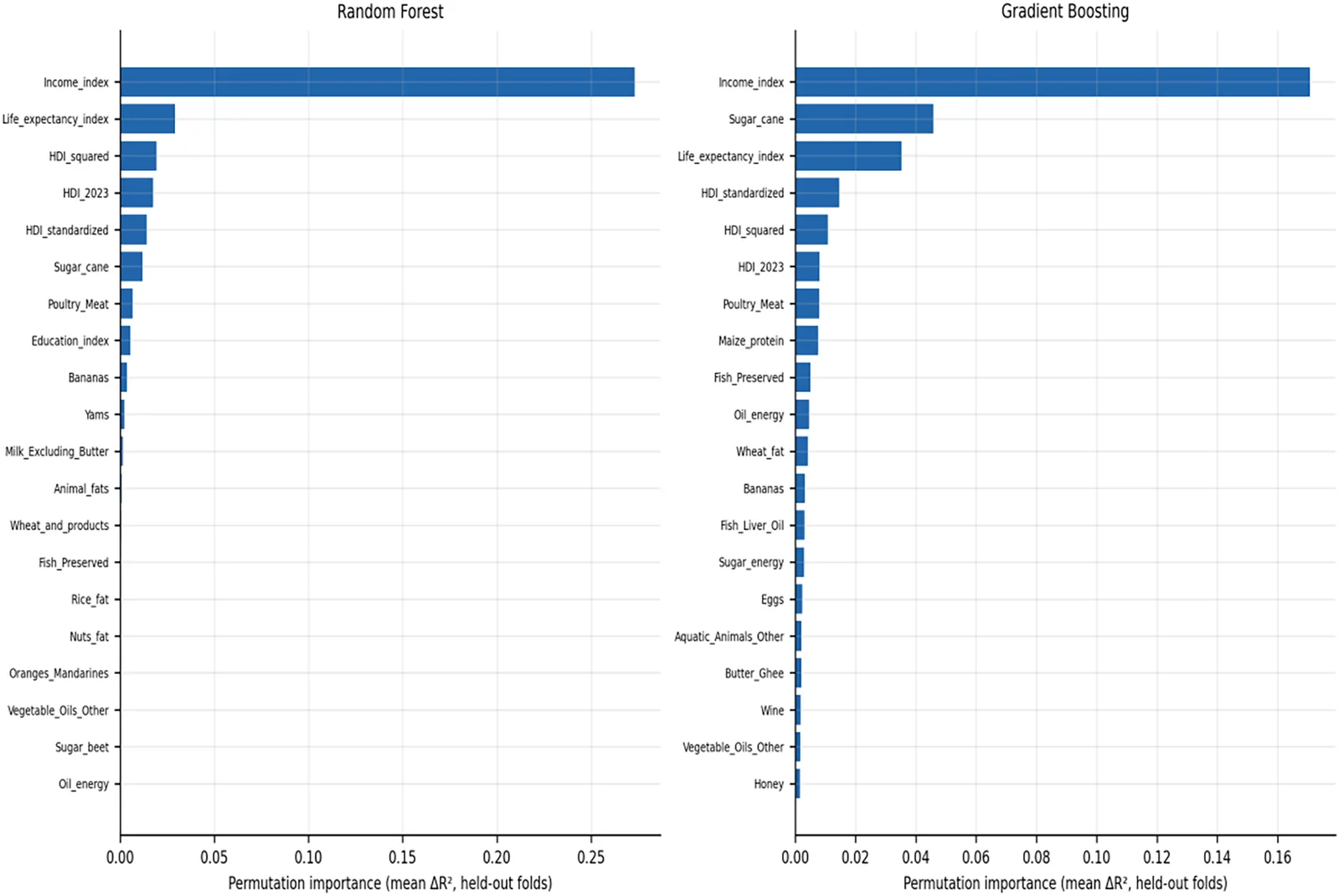
Permutation importance for the top 20 features (mean Δ*R*² over 30 permutation repeats), computed exclusively on held-out folds and averaged across folds, for random forest (left) and gradient boosting (right). The ranking is dominated by socioeconomic sub-indices; rankings are also sensitive to collinearity among predictors and should not be read as evidence about etiology.

### 3.4 Dietary factors associated with incidence after confounder adjustment

The third objective was to identify which dietary factors remain associated with incidence once development and metabolic confounding are removed. The Spearman correlation matrix (**Figure 6**) first describes the joint structure of cancer burden, dietary supply, and socioeconomic indicators. Incidence correlated most strongly with HDI (*ρ* = 0.912) and with the life expectancy index (*ρ* = 0.875), a far stronger development gradient than any dietary variable produces, followed by the iodine-rich food availability index (*ρ* = 0.234), total energy supply (*ρ* = 0.230), the animal-to-plant protein ratio (*ρ* = 0.221), seafood supply (*ρ* = 0.219), animal protein (*ρ* = 0.190), mollusks (*ρ* = 0.187), dairy supply (*ρ* = 0.176), and obesity prevalence (*ρ* = 0.170). Plant protein supply was inversely associated (*ρ* = −0.167). The dominant feature of this matrix is that the dietary variables are themselves closely tied to HDI, which makes confounder-adjusted analysis indispensable rather than confirmatory. Unadjusted associations for the nine principal dietary and metabolic variables, with quadratic fits, are shown in **Supplementary Figure S2**, and the same variables stratified by UNDP human development category are shown in **Supplementary Figure S3**; the within-group spread of incidence was widest in the very high category, so development level alone does not account for the heterogeneity observed among wealthy countries. Principal component and cluster analyses of the national food supply matrix (**Supplementary Figures S4**–**S6**) reproduce the familiar contrast between affluent and staple-crop dietary patterns, but the principal dietary axis tracks HDI closely, and the clusters differ in development at least as much as in diet, so neither can be read as a dietary effect.

**Figure 6:**
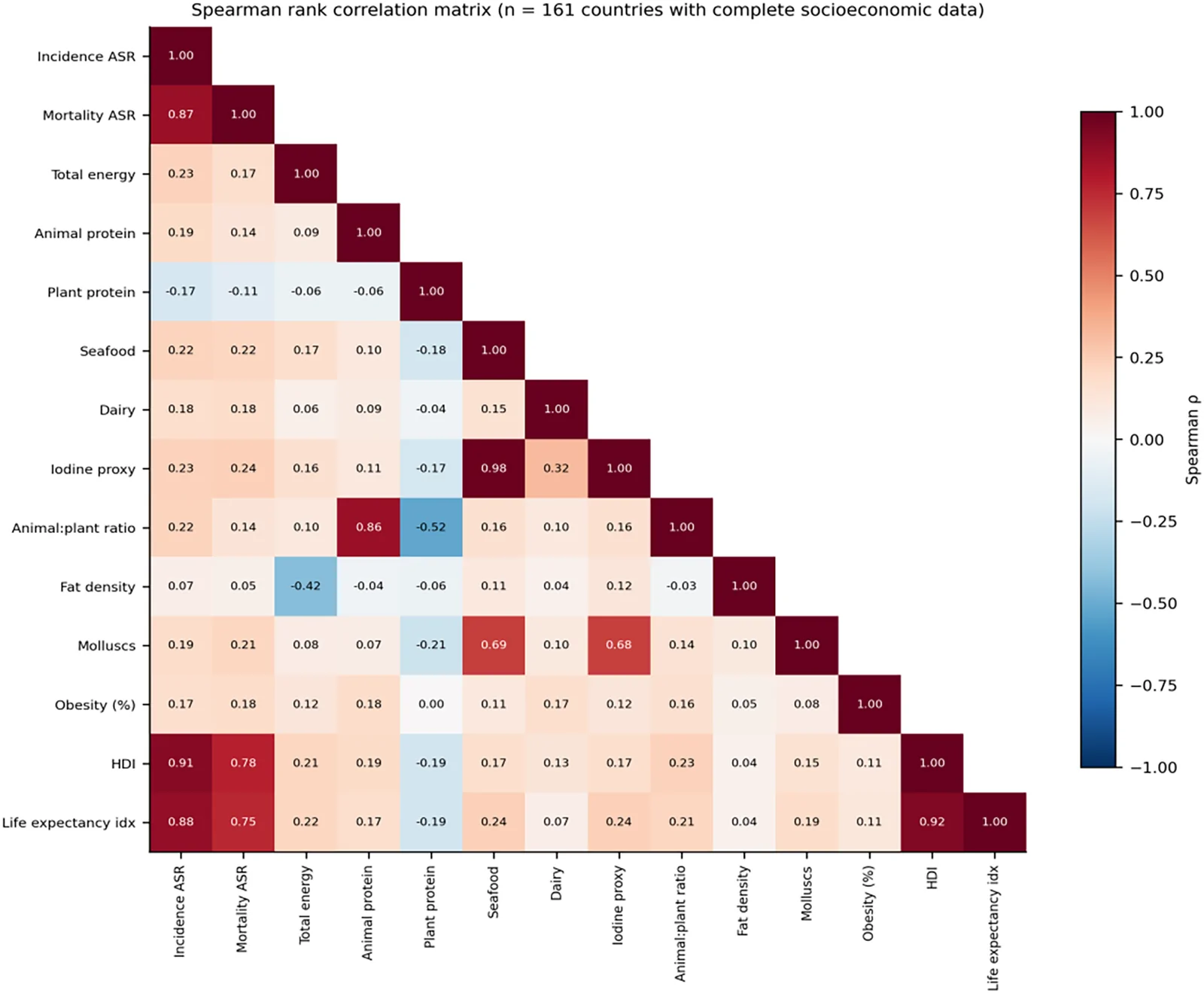
Spearman rank correlation matrix (lower triangle) among thyroid cancer burden and dietary and socioeconomic variables, with coefficients printed in each cell. Red indicates positive and blue indicates negative correlations. The close correlation of dietary variables with HDI is the reason confounder-adjusted analysis is required. *n* = 161 countries with complete socioeconomic data, matching the sample used in **Table 2**.

**Table 2** reports all three association models side by side, and **Figure 7** displays them. Before adjustment, most dietary variables were significantly associated with incidence after FDR correction. Adjustment for HDI and obesity attenuated these associations, and the attenuation was strongly variable-specific rather than uniform: the variables that track total supply or affluence lost almost all of their association—total energy fell from *ρ* = 0.230 to a partial *r* of 0.098 (*q* = 0.309), the Western diet score from 0.181 to 0.043, and obesity prevalence from 0.170 to 0.020—whereas the iodine-related variables were barely attenuated at all. Under the basic adjustment set, seafood supply, the iodine-rich food availability index, and the animal-to-plant protein ratio remained significant after Benjamini–Hochberg correction. Under the extended set, which adds the three HDI sub-indices and region fixed effects, seafood supply, dairy supply, the iodine-rich food availability index, the animal-to-plant protein ratio, and mollusks remained significant, with partial correlations of 0.260 for the animal-to-plant protein ratio (*q* = 0.011), 0.224 for the iodine-rich food availability index (*q* = 0.026), 0.203 for mollusks (*q* = 0.031), 0.198 for dairy supply (*q* = 0.031), and 0.196 for seafood supply (*q* = 0.031). That these associations survive both the addition of health-system and geographic proxies and correction for multiple comparisons is the central positive finding of this study. Plant protein does not: its partial correlation was −0.095 (uncorrected *p* = 0.232, *q* = 0.309) under basic adjustment and –0.149 (*q* = 0.088) under extended adjustment, and we report it as a null result rather than as a protective association. The Bonferroni-adjusted values for all three families are given in **Supplementary Table S6**; under that more conservative correction, the animal-to-plant protein ratio remains significant in both adjusted families and the iodine-rich food availability index in the basic family (*p* = 0.038), with the extended-family value being borderline (*p* = 0.052). The sensitivity of the seafood association to the exposure window (0–12 years) is reported in **Supplementary Figure S13**: the correlation varied only between *ρ* = 0.213 and *ρ* = 0.232 with no trend toward longer lags, and because national food supply is highly autocorrelated, these windows are not independent tests and do not establish temporal precedence.

**Figure 7:**
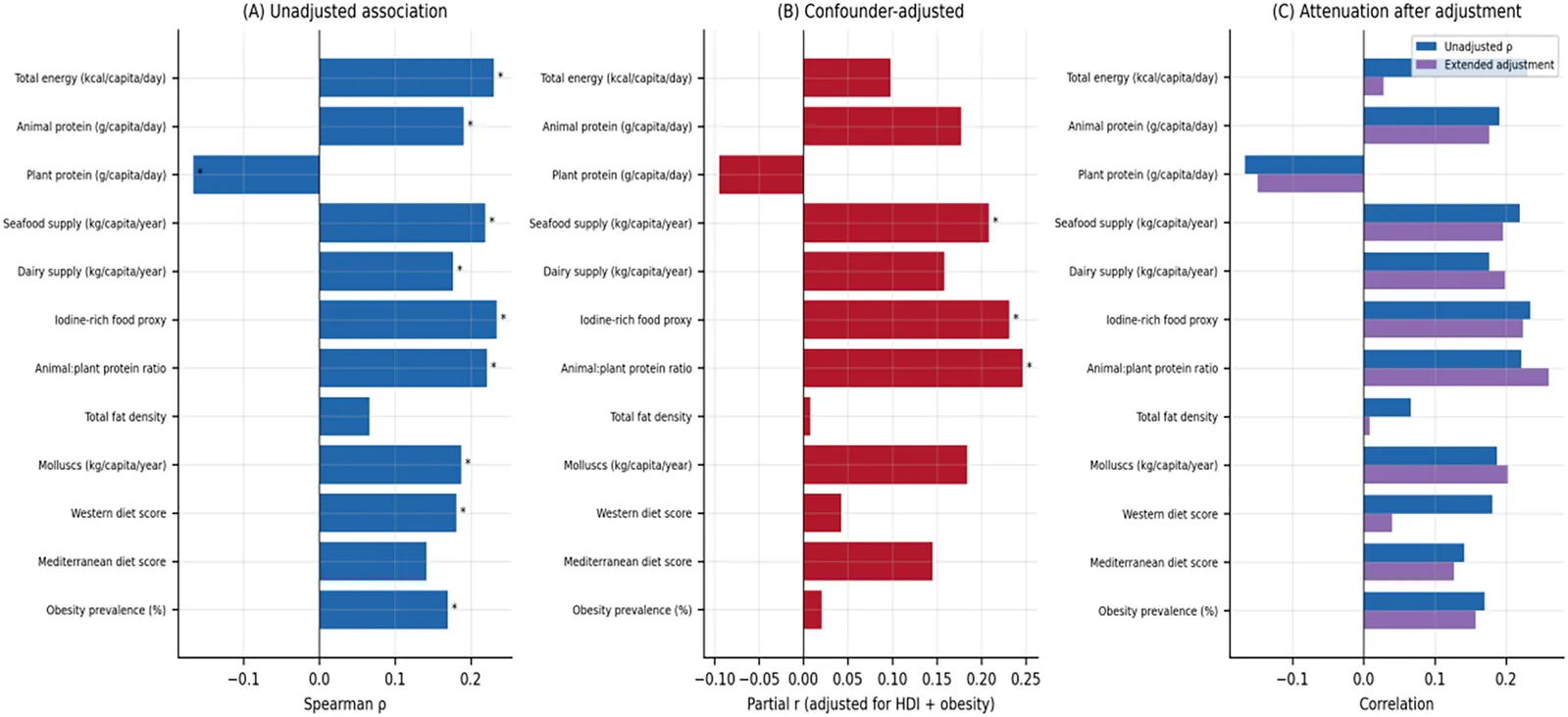
Comparison of bivariate and confounder-adjusted associations between dietary factors and thyroid cancer incidence. **(A)** Bivariate Spearman correlations. **(B)** Partial correlations adjusted for HDI and obesity. **(C)** Side-by-side comparison showing confounding attenuation. Asterisks indicate statistical significance (*p < 0.05, **p < 0.01). Sensitivity analyses for the iodine-rich food availability index are reported in **Table 3** and **Supplementary Figure S16**.

### 3.5 Sensitivity analyses for the iodine-rich food availability index

Because the index is a weighted composite of three food groups and measures availability rather than intake, four prespecified sensitivity analyses interrogated its construction (Section 2.7.2). The results are summarized in **Table 3** and displayed in **Supplementary Figure S16**, with the full output displayed in **Supplementary Tables S13**–**S16**. Entered separately in place of the composite, seafood supply (partial *r* = 0.196, *q* = 0.017) and dairy supply (0.198, *q* = 0.017) each retained a positive association with incidence under the extended adjustment set, whereas egg supply did not (0.065, *q* = 0.411) despite a comparable unadjusted correlation (*ρ* = 0.217). The index association is therefore carried by the two food groups that dominate its variance and that are the principal dietary sources of iodine in most populations and not by its smallest component. Entered simultaneously as standardized terms in a single multivariable model, seafood and dairy had similar coefficients net of one another under extended adjustment (*β* = 0.476, *p* = 0.052 and *β* = 0.478, *p* = 0.043 per SD), while eggs did not (*β* = 0.106, *p* = 0.657); variance inflation factors were 1.03–1.07, and the pairwise Spearman correlations among the three components were 0.045–0.184, so the components are close to orthogonal and the composite is not double-counting a single underlying supply pattern (**Supplementary Table S14**).

The conclusion does not depend on how the three components are weighted. Across the primary construction, an equal-weighted sum of raw supply quantities, an equal-weighted sum of standardized components, an iodine-content-weighted sum, and a seafood-only variant, the extended-adjustment partial correlation ranged from 0.196 to 0.256, and every variant survived Benjamini–Hochberg correction (*q* ≤ 0.013). The equal-weighted and iodine-content-weighted constructions gave slightly larger estimates than the primary index (0.250 and 0.256 versus 0.224), so the weighting used in the main analysis is, if anything, conservative. Pairwise Spearman correlations among the five variants ranged from 0.56 to 0.98, confirming that they order countries similarly (**Supplementary Table S15**).

The fourth analysis addressed whether the index reflects animal-origin food availability in general rather than iodine-rich food availability specifically. Adding total animal protein supply to the extended adjustment set left the index association essentially unchanged (partial *r* = 0.203, *q* = 0.032), which is unsurprising given that the two variables are only weakly related across countries (*ρ* = 0.109). By contrast, an iodine-poor animal-origin food index built from bovine, pig, poultry, and mutton and goat meat had the largest unadjusted correlation of any variable examined in this analysis (*ρ* = 0.309) but did not survive adjustment (partial *r* = 0.135, *q* = 0.087 basic; 0.102, *q* = 0.198 extended; 0.088, *q* = 0.537 with animal protein added), and total animal products supply behaved likewise once animal protein was in the adjustment set (0.024, *q* = 0.961). Total animal protein supply itself retained a modest association under extended adjustment (0.176, *q* = 0.035), so we do not claim that animal-origin food availability is unrelated to incidence; what these analyses show is that the association of the iodine-rich food availability index is not explained by it and that the animal-origin food groups poorest in iodine are precisely the ones whose association does not survive confounder adjustment (**Supplementary Table S16**). We nonetheless emphasize that this pattern is consistent with an iodine-related interpretation without establishing one and that the index remains a measure of national food-group availability throughout.

### 3.6 Contribution of the development pathway to the seafood–incidence association

The fourth objective was to quantify how much of the ecological seafood–incidence association tracks national development. The descriptive path decomposition (**Figure 8**) partitions that correlation into a component that tracks HDI and a residual component. The total association was *c* = 0.1149; the HDI-tracking component (*a* × *b*) was 0.0576, corresponding to 50.2% of the total, with a residual direct term of *c*′ = 0.0573. The bootstrap interval for that percentage runs from 16% to 90%, so the point estimate should be read as “roughly half, with wide uncertainty” rather than as a precise partition. Two things follow. Development status accounts for a large share of the ecological correlation between national seafood supply and thyroid cancer incidence, which is what the overdiagnosis literature would predict. However, a residual component remains, and it is consistent with the adjusted association analysis, in which seafood retains a positive partial correlation after adjustment for HDI, its sub-indices, obesity, and region (Section 3.4). We nevertheless do not interpret the residual as evidence of an iodine-mediated pathway: it is a cross-sectional ecological quantity, and unmeasured confounders including radiation exposure history, national iodization programs, and genetic susceptibility remain plausible explanations.

**Figure 8:**
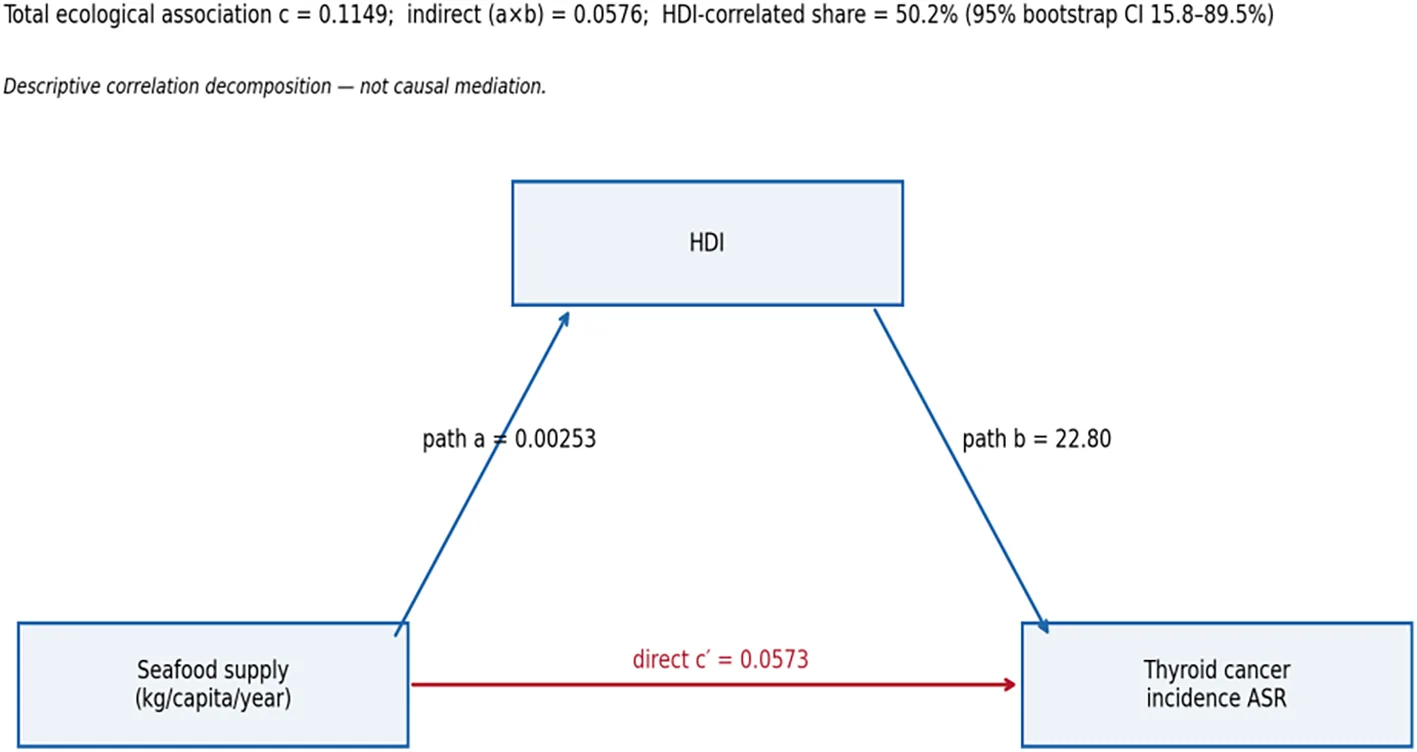
Descriptive decomposition of the ecological seafood–incidence correlation through the HDI pathway. Path coefficients are shown on each arrow. The HDI-tracking component accounts for 50.2% of the total association, with a 95% bootstrap interval of 16%–90% (5,000 resamples of countries). This is a correlation decomposition, not a causal mediation analysis.

### 3.7 Secondary analysis: mortality and differential cancer detection

Mortality enters this study only at this point and only for the purpose set out in Section 2.7.5: to assess whether the incidence patterns described above are attributable to differential cancer detection. **Figure 9** compares associations across three outcomes—incidence, mortality, and the mortality-to-incidence ratio. HDI was the strongest correlate of both incidence (*ρ* = 0.912) and mortality (*ρ* = 0.778), and the dietary variables followed the same ordering for both outcomes, so the incidence associations are not a pattern that appears only in a detection-sensitive outcome. Associations with the mortality-to-incidence ratio, by contrast, were close to zero or negative (HDI ρ = 0.053; seafood *ρ* = 0.065). Since that ratio is comparatively insensitive to detection intensity while incidence is not, this contrast indicates that the ecological correlations described in this study operate substantially through case detection rather than through case fatality. This is a check on the interpretation of the primary incidence findings, not an independent result: no dietary conclusion of this study is drawn from the mortality data, and mortality is not modeled or decomposed anywhere in this manuscript.

**Figure 9:**
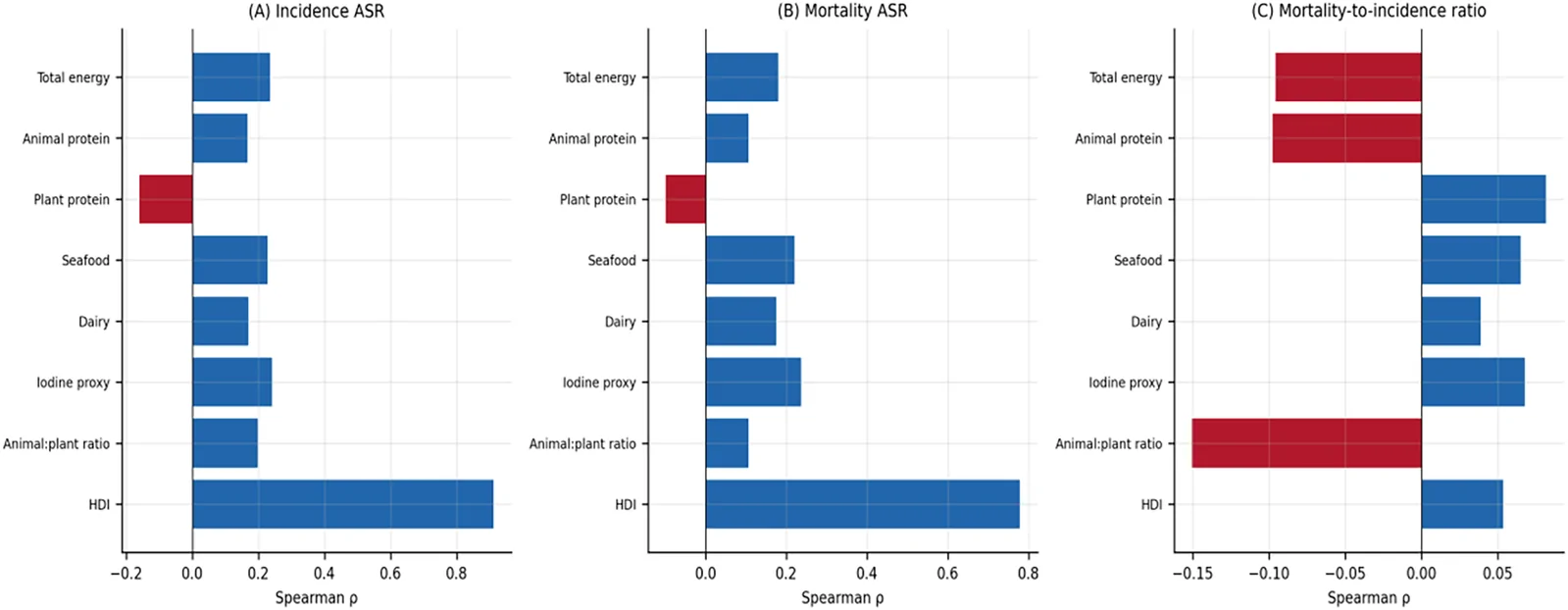
Secondary analysis of differential cancer detection: multi-target Spearman correlations of eight dietary and socioeconomic predictors with **(A)** incidence ASR, the primary outcome, **(B)** mortality ASR, and **(C)** the mortality-to-incidence ratio across 163 countries. Associations with the ratio are close to zero, consistent with the interpretation that these ecological correlations act substantially on case detection rather than on case fatality.

### 3.8 Secondary and exploratory analyses

The remaining analyses are secondary or exploratory and are reported in full in the **Supplementary Material** so that the main text can remain focused on the four questions above. They comprise the distribution and transformation of the outcome variables (**Supplementary Figure S1**); unadjusted dietary scatterplots and HDI-stratified comparisons (**Supplementary Figures S2**, **S3**); principal component and K-means analyses of national dietary patterns (**Supplementary Figures S4**–**S6**); observed versus out-of-fold predicted rates for the four leading models (**Supplementary Figure S7**); the binary classification sensitivity analysis, in which Extra Trees reached AUC = 0.932 (95% CI [0.893, 0.964]) at the median split and random forest AUC = 0.985 at the upper-quartile threshold, a rise that illustrates why these figures should not be read as evidence of clinical utility since the further apart the two groups are placed on a skewed continuous scale the easier the contrast becomes (**Supplementary Table S12**; **Supplementary Figures S8**, **S9**); partial dependence, contribution, and development-stratified explainability plots (**Supplementary Figures S10**–**S12**); the temporal lag sensitivity profile (**Supplementary Figure S13**); residual diagnostics for a leading model, which show no obvious curvature but increasing spread with predicted incidence (**Supplementary Figure S14**); and leave-one-region-out validation (**Supplementary Figure S15**). None of these analyses supports a conclusion that is not already established in Sections 3.2–3.6.

## 4 Discussion

### 4.1 Summary of principal findings

Four findings emerged, all of them concerning thyroid cancer incidence. First, with all model construction confined to training folds, the best model (stacking ensemble) reached a pooled out-of-fold *R*² of 0.613 (95% CI [0.510, 0.726]) with Spearman *ρ* = 0.901, and the advantage of ensembles over regularized linear models was small. Second, that accuracy is socioeconomic rather than dietary: nine development variables alone achieved *R*² = 0.609 and 140 dietary variables alone 0.284, and the incremental contribution of the dietary block was indistinguishable from zero in all three model families. Third and in contrast, several dietary associations did survive both confounder adjustment and correction for multiple comparisons: seafood supply, dairy supply, the iodine-rich food availability index, the animal-to-plant protein ratio, and mollusks retained positive partial correlations under an adjustment set containing HDI, its three sub-indices, obesity prevalence, and region fixed effects, with the largest for the animal-to-plant protein ratio (0.260, *q* = 0.011) and the iodine-rich food availability index (0.224, *q* = 0.026), while plant protein showed no association once multiplicity was controlled. Sensitivity analyses located the index association in seafood and dairy availability, showed it to be insensitive to the weighting of the composite, and showed that it is not reproduced by iodine-poor animal-origin foods. Fourth, roughly half of the seafood–incidence correlation tracks the development pathway (50.2%, 95% bootstrap CI 16–90%), and region-held-out validation shows that the models transport the ordering of countries but not their level. The secondary mortality analysis is consistent with all of this: dietary variables order similarly for mortality as for incidence, while associations with the mortality-to-incidence ratio are close to zero. Read together, these results describe an ecological pattern in which national development and detection intensity dominate the predictive signal, while a specific and multiplicity-robust association between the availability of iodine-dense animal-origin food groups and incidence persists after adjustment.

### 4.2 Comparison with previous studies

Comparing performance metrics across studies with different outcomes, predictors, and units of analysis has limited value, and we make such comparisons only to situate the present results. Abdul Rahman et al. (20) reported accuracy above 0.90 for colorectal cancer prediction from dietary data, and Guo et al. (19) AUCs of 0.78–0.86 for breast cancer incidence from biochemical biomarkers; both differ from the present study in outcome, predictors, and unit of analysis. We are not aware of a directly comparable benchmark for our regression R^2^ of 0.613, and any comparison would be weak: our unit of analysis is the country, and as Section 3.3 shows, most of the explained variance is socioeconomic. The classification AUC of 0.932 reported in the sensitivity analysis (**Supplementary Table S12**) should not be compared with individual-level AUCs at all since a median split of 163 country-level rates is a far coarser discrimination problem than classifying individuals.

The positive association between the availability of iodine-rich food groups and thyroid cancer incidence is the most robust finding of this study. It survives adjustment for HDI and obesity, further adjustment for the three HDI sub-indices and region fixed effects, further adjustment for total animal protein supply, and Benjamini–Hochberg correction across all 12 variables in each family (partial *r* = 0.224, *q* = 0.026 under extended adjustment). It is also robust to the construction of the index itself: it is present for seafood and for dairy entered separately, for four different weighting schemes, and for a seafood-only variant. Individual-level evidence does not straightforwardly corroborate it: evidence on iodine intake and thyroid cancer risk is inconsistent (10), a meta-analysis of 17 studies found no association between seafood intake and thyroid cancer risk (11), and a recent meta-analysis reported a lower risk with higher fish consumption (14). The comparison must be made carefully: those studies measured intake in individuals, whereas our estimate is a between-country association in the availability of food groups that are rich in iodine. What our analysis adds is that this particular association is not an artifact of the two problems that most plausibly generate spurious ecological diet findings—confounding by development and uncorrected multiplicity—because it withstands both.

The inverse association between plant protein supply and incidence did not persist under adjustment and multiplicity correction (partial *r* = −0.095, uncorrected *p* = 0.232, *q* = 0.309 under basic adjustment; −0.149, *q* = 0.088 under extended adjustment), and we therefore report it as a null result. We state this plainly because a mechanistic interpretation invoking isoflavones, thyroid peroxidase inhibition, and estrogen receptor signaling would rest on an estimate that is significant only before multiplicity correction. Individual-level evidence is limited and inconsistent: a recent systematic review associated plant-rich dietary patterns with a lower thyroid cancer risk (45), whereas a prospective cohort found no significant association for frequent fruit intake (12), and the present study adds no independent support for it, and we offer no mechanistic account of a null result. We note that the direction of the estimate is consistently negative under both adjustment sets and that the extended-adjustment *q*-value (0.088) is close to the conventional threshold, which makes plant protein a reasonable candidate for individual-level testing rather than a finding of this study.

Mollusks remain of interest, but not because of the model importance rankings, which are dominated by the income and life expectancy sub-indices (Section 3.3). Their standing rests instead on the confounder-adjusted association analysis, where mollusk supply retains a partial correlation of 0.203 (*q* = 0.031) under extended adjustment. These food groups are among the richest dietary sources of iodine—a 3-oz (85 g) serving of cooked oysters provides about 93 µg of iodine (46)—so their persistence after adjustment, alongside the broader iodine-rich food availability index and the animal-to-plant protein ratio, is consistent with an iodine-related hypothesis. It does not establish one. Testing it requires individual-level designs with measured urinary iodine concentrations, and we present it as a hypothesis that these data sharpen rather than as a mechanism they support.

#### 4.2.1 Model class and recent tabular foundation models

The shape of this prediction problem—163 observations, 150 correlated predictors, and a strongly skewed outcome—is the regime in which conventional gradient-boosted and bagged ensembles are known to struggle and in which a rapidly developing family of tabular foundation models has been proposed as an alternative. Transformer-based in-context learners such as TabPFN-3 (47) are pre-trained on large collections of synthetic tabular tasks and predict without per-dataset gradient training, which is attractive at this sample size. Related work addresses the high-dimensional case directly: GOTabPFN reduces high-dimensional inputs through feature ordering and compact tokenization (48), DynaTab treats feature ordering as a dynamic rewiring problem (49), and TabSeq imposes a sequential ordering on features before deep sequence modeling (50). We therefore benchmarked DynaTab (49), the reference among these methods, built specifically for high-dimensional low-sample-size regression and requiring no external pretrained checkpoint, against our existing estimators under the identical nested cross-validation protocol (**Table 1**). DynaTab was applied as a drop-in estimator, with multiple imputation, standardization, and the four-method consensus selection refitted inside every training fold; because it is a neural model on a small sample, over-fitting was controlled by a leakage-free internal validation split with best-epoch restoration and the initialization variance of such a model at this sample size by seed-ensembling (three independently initialized members per fold, their predictions averaged), with all hyper-parameters fixed *a priori*. DynaTab attained a pooled out-of-fold *R*² of 0.626 (95% CI [0.539, 0.727]), statistically comparable to the strongest conventional models—the stacking ensemble (0.613) and random forest (0.609)—with intervals that overlap almost entirely and a rank correlation (*ρ* = 0.888) and RMSE (2.99) in line with the ensembles; the estimate is reproducible, giving *R*² = 0.609 and 0.606 across the two fold partitions and 0.624 under an independent re-run with different seeds, whereas a single-seed run varied between 0.52 and 0.64. A purpose-built high-dimensional model therefore matches, rather than overturns, the predictive accuracy achievable on these data, and it does not alter the block-wise finding of Section 3.3, which concerns which variable groups carry the between-country signal—development indicators rather than diet—rather than which model extracts it; a preliminary evaluation on the full 150-variable matrix without selection placed DynaTab below its selected-feature accuracy, indicating that, unlike the tree ensembles, the neural model benefits from the prior feature-selection step. We did not benchmark TabPFN-3, GOTabPFN, or TabSeq here—the first two are a pretrained tabular foundation model and a wrapper around it, and TabSeq targets classification rather than our primary continuous task, but the released **Supplementary Code S1** exposes the estimator list as a single configuration block, so any of them can be evaluated under the same protocol with no other change, which we identify as the most concrete methodological extension of the present work.

### 4.3 Decomposing the seafood–incidence correlation through the development gradient

The path decomposition quantifies how far the ecological seafood–incidence correlation follows the development gradient. The HDI-tracking component accounted for 50.2% of the total association (path a = 0.00253; path b = 22.80), which is consistent with the well-documented detection pathway in which greater national wealth brings more thyroid imaging and more incidental cancers (8, 51). We report the bootstrap interval for that share, 16%–90%, so that the point estimate is not over-read. The residual direct term (*c*′ = 0.0573) is consistent with the adjusted association analysis, in which seafood supply retains a positive partial correlation after adjustment for HDI, its sub-indices, obesity, and region. We nonetheless make no causal claim about it. The analysis is a descriptive decomposition of a cross-sectional ecological correlation; it satisfies neither the temporal nor the counterfactual requirements of causal mediation. Whether a biological iodine pathway exists is a question for individual-level designs with measured urinary iodine concentrations.

### 4.4 What the iodine-rich food availability index does and does not measure

A clear statement of what this variable measures is necessary for the interpretation of our most robust finding. The index is constructed from national per-capita food supply of seafood, dairy, and eggs. It is therefore a measure of the availability of iodine-rich food groups in a national food supply, and it is not a measure of iodine intake, of iodine status, or of iodine exposure in any individual. Three distinct gaps separate it from iodine exposure. First, food balance sheets describe supply rather than consumption and do not capture waste, spoilage, or unequal distribution within a country. Second, the iodine content of a given food group varies substantially with production method, soil and water iodine content, feed composition, processing, and, for dairy in particular, the use of iodine-containing teat disinfectants; a kilogram of milk in one country is not nutritionally equivalent to a kilogram in another. Third and most important at the population level, the largest single determinant of iodine intake in most countries is not any of these food groups but salt iodization, which FAOSTAT does not measure and which we could not adjust for at the country level. For all three reasons, we describe our result throughout as an association between the availability of iodine-rich food groups and thyroid cancer incidence, and we do not describe it as an association with iodine exposure. The weighting of the composite is likewise an analytical choice rather than a measurement, which is why we report it explicitly (seafood + 0.10 × dairy + 0.05 × eggs), why we tested equal-weighted, standardized, and iodine-content-weighted alternatives, and why we report the components separately as well as jointly. Those analyses (Section 3.5) support three specific statements and no more: the association is present for seafood and for dairy availability considered separately, it does not depend on the weighting scheme, and it is not reproduced by the animal-origin food groups that are poorest in iodine nor removed by adjustment for total animal protein supply. That last comparison is the strongest argument available in these data against the alternative reading of the index as a marker of animal-origin food availability in general, but it is an argument from an ecological negative control, not a demonstration of an iodine mechanism, and it cannot exclude residual confounding by correlates of seafood and dairy availability such as coastal geography, national iodization policy, or the intensity of thyroid imaging.

### 4.5 Methodological strengths

The strengths of this work lie in its data integration and in the conservatism of its analysis. Four open databases were harmonized into a single country-level file covering 163 countries, with the socioeconomic block extracted from the primary UNDP source and verified against the published index. All model construction is nested inside cross-validation; confidence intervals are obtained by resampling countries, the actual units of analysis; the incremental contribution of dietary information is quantified explicitly against a socioeconomic baseline; generalization is tested across held-out world regions as well as random folds, with rank agreement reported alongside *R*²; multiplicity is controlled across all association families under two corrections; the construction of the principal exposure variable is examined component by component and under alternative weightings, with an iodine-poor negative control; and the complete pipeline, including the seeds and the socioeconomic reconstruction, is released so that every number can be reproduced or contradicted. The analysis also reports results that run against our own prespecified expectations.

### 4.6 Limitations

Several limitations bound what these data can support. The design is ecological, so country-level associations cannot be transferred to individuals; every estimate reported here is a property of countries. FAOSTAT food balance sheets describe national supply rather than intake and do not capture waste, spoilage, or unequal distribution, which is why we use the term “food supply availability” throughout. GLOBOCAN estimates are partially modeled for countries without population-based registries, so incidence is measured with error that is itself correlated with development. Residual confounding by diagnostic intensity remains the central threat to interpretation and is not resolved by the adjustments used here: HDI, its sub-indices, and region indicators are proxies, and no global source provides country-level thyroid ultrasound utilization, fine-needle aspiration rates or screening policy, which are the variables that would be required. The underprediction of Cyprus and the Republic of Korea and the near-zero associations with the mortality-to-incidence ratio both point to this unmeasured pathway. FAOSTAT provides no iodine-specific nutrient data, so iodine-related exposure is represented by the availability of food groups rather than by measured intake, and the iodine interpretation of our most robust association is therefore indirect for the reasons set out in Section 4.4; salt iodization, the dominant determinant of population iodine status in most countries, could not be adjusted for. The secondary mortality analysis is likewise indirect: mortality-to-incidence ratios are influenced by treatment access and by case-fatality reporting as well as by detection, and they provide a consistency check rather than a formal test of overdiagnosis. Exposure (2015–2019) and outcome (2022) windows are mismatched, and the design cannot establish temporal precedence; the lag analyses use highly autocorrelated data and are not independent replications. Extensive collinearity among predictors makes individual feature importance rankings unstable. HDI was unavailable for two French overseas collectivities and was imputed within training folds, so 161 rather than 163 countries contribute to the confounder-adjusted association analyses. With 163 countries, power is limited: bootstrap intervals around *R*² remain wide, region-held-out performance is estimated from as few as 10 countries in one region, and the path decomposition ratio carries a wide interval. No external country-level dataset was available for validation, so generalizability beyond this sample is untested.

### 4.7 Implications for public health and future research

Two implications follow, one negative and one positive. The negative one is methodological: in these data, adding 140 dietary variables to nine development indicators produced no improvement in prediction that the bootstrap intervals can distinguish from zero (Δ*R*² between −0.048 and +0.021, all intervals spanning zero), while nine development indicators alone reached *R*² = 0.609, and the model importance rankings are dominated by the income and life expectancy sub-indices. Ecological machine learning studies that report dietary predictors of cancer burden without an explicit socioeconomic baseline, a leakage-free validation design, and multiplicity control risk attributing to diet what belongs to development. The positive one concerns the specific hypothesis this study was designed to test: the availability of iodine-dense animal-origin food groups—the iodine-rich food availability index, seafood, mollusks, dairy, and the animal-to-plant protein ratio—retains a positive association with thyroid cancer incidence after adjustment for development, metabolic status, and geography, after correction for multiple comparisons, and after the construction of the index is varied and tested against an iodine-poor negative control. That is a hypothesis worth taking to designs that can test it: cohorts with validated dietary assessment and measured urinary iodine (UK Biobank, EPIC, and NIH-AARP), direct measures of national thyroid imaging utilization and salt iodization coverage to separate detection and iodine policy from food-group availability, Mendelian randomization to address confounding by design, genomic data such as TCGA-THCA for gene–diet interaction, and longitudinal panel models in which exposure is observed before outcome. Until such evidence exists, these findings remain ecological correlates and should not be translated into individual dietary advice.

## 5 Conclusions

With feature selection, imputation, scaling, and tuning performed inside cross-validation folds, machine learning models built from national food supply and socioeconomic data explained a moderate share of the between-country variation in thyroid cancer incidence, the prespecified primary outcome (best pooled out-of-fold *R*² = 0.613, 95% CI [0.510, 0.726]; Spearman *ρ* = 0.901), with only a small advantage for ensembles over regularized linear models. Block-wise analysis localizes that accuracy in development indicators rather than in diet: nine socioeconomic variables achieved *R*² = 0.609 on their own, and adding 140 dietary variables changed *R*² by an amount indistinguishable from zero in every model family tested. The dietary findings of this study therefore rest on association rather than on prediction and there they hold: seafood supply, dairy supply, the iodine-rich food availability index, the animal-to-plant protein ratio, and mollusks retained positive partial correlations with incidence after adjustment for HDI, its three sub-indices, obesity prevalence, and region fixed effects and after Benjamini–Hochberg correction across all comparisons, while plant protein showed no association once multiplicity was controlled. Sensitivity analyses show that the index association is carried by seafood and dairy availability, is insensitive to how the composite is weighted, survives adjustment for total animal protein supply, and is not reproduced by iodine-poor animal-origin foods; it is, nevertheless, an association with the availability of iodine-rich food groups and not with measured iodine intake. Approximately half of the seafood–incidence correlation tracks the development pathway (50.2%, 95% CI 16–90%), models transport the ordering of countries across held-out regions better than their level, and the secondary mortality analysis is consistent with a substantial contribution from differential detection. The contribution of this work is a leakage-free, multiplicity-controlled quantification of how strongly national development dominates ecological diet–thyroid cancer patterns, together with one dietary signal that survives that scrutiny and a fully released pipeline with which the analysis can be repeated, extended, or refuted.

## Data Availability

The original contributions presented in the study are included in the article/**Supplementary Material** (**Supplementary Data S1** and **Supplementary Code S1**); further inquiries can be directed to the corresponding author.
