# Supplementary material for: Global thyroid cancer incidence in relation to dietary and socioeconomic factors: an ecological machine learning study
Source: Front Oncol. 2026 Oct 16;16:1842508. doi: 10.3389/fonc.2026.1842508 (PMC13719374; doi:10.3389/fonc.2026.1842508)
Supplement: Supplementary file 1 [file DataSheet1.zip › Supplementary Material S1-S16.docx]

**Supplementary Material**

**Global Thyroid Cancer Incidence in Relation to Dietary and Socioeconomic Factors: An Ecological Machine Learning Study**

Li Yixing, Liu Dehui, Qin Ruixue, Li Xingchang, Li Mei

**Contents**

Supplementary Table S1. TRIPOD-AI reporting checklist

Supplementary Table S2. STROBE (ecological) reporting checklist

Supplementary Table S3. Sex-stratified association analysis

Supplementary Table S4. Multiple-imputation (MICE) sensitivity analysis

Supplementary Table S5. Complete variable dictionary (150 numeric predictors)

Supplementary Table S6. Unadjusted and confounder-adjusted associations with FDR and Bonferroni correction

Supplementary Table S7. Feature-selection stability across folds

Supplementary Table S8. Fold-level variability of regression performance

Supplementary Table S9. Model performance without any feature-selection step

Supplementary Table S10. Block-wise models and the incremental predictive value of dietary features

Supplementary Table S11. Leave-one-region-out validation

Supplementary Table S12. Classification sensitivity analysis at two thresholds

Supplementary Table S13. Individual components of the iodine-rich food availability index

Supplementary Table S14. Joint multivariable model of the three index components

Supplementary Table S15. Alternative weighting schemes for the index

Supplementary Table S16. Specificity: iodine-rich versus iodine-poor animal-origin food availability

Supplementary Figures S1–S15. Secondary and exploratory analyses relocated from the main text

Supplementary Figure S16. Sensitivity analyses for the iodine-rich food availability index

Supplementary Data S1. Harmonised country-level analytical dataset (CSV and XLSX) with variable dictionary

Supplementary Code S1. Complete executable analysis pipeline (Python, seed 42), Parts 0–4

*Supplementary Tables S13–S16 and Supplementary Figure S16 are new. Supplementary Figures S1–S15 present the secondary and exploratory analyses that have been relocated from the main text so that the main manuscript can be organised around its four primary research questions. All other supplementary items are unchanged.*

**Supplementary Table S1. TRIPOD-AI reporting checklist**

| **Item** | **Reporting element** | **Manuscript location** |
| --- | --- | --- |
| 1 | Title identifies study as ML prediction model | Title |
| 2 | Structured abstract of objectives, methods, results | Abstract |
| 3a | Background and rationale | Introduction |
| 3b | Objectives / pre-specified hypotheses | Introduction (final paragraph) |
| 4a | Source of data (GLOBOCAN, FAOSTAT, UNDP, WHO) | §2.2 |
| 4b | Dates / exposure windows | §2.2.2, §2.4.4 |
| 5a | Study setting: 163 countries, ecological | §2.1, §2.3.1 |
| 6a | Outcome definition (incidence ASR) | §2.2.1 |
| 7a | Predictors and feature engineering | §2.4 |
| 8 | Sample size (all countries with data) | §2.3.1 |
| 9 | Missing data (MICE within folds) | §2.3.2, Table S4 |
| 10a | Preprocessing / standardization within folds | §2.3.3 |
| 10b | Model specification (13 regressors) | §2.6.1 |
| 10c | Nested cross-validation / tuning | §2.6.1 |
| 10d | Performance measures (pooled OOF, bootstrap CI) | §2.6.1 |
| 11 | Risk groups (median split, sensitivity only) | §2.6.2 |
| 15a | Model performance | §3.5, Table 2 |
| 16 | Explainability (held-out permutation, PDP) | §2.8, §3.7 |
| 19b | Interpretation / limitations | §4.1–4.6 |
| 20 | Data and code availability | §2.9, Data Availability |

**Supplementary Table S2. STROBE (ecological) reporting checklist**

| **Item** | **Reporting element** | **Manuscript location** |
| --- | --- | --- |
| 1 | Study design in title/abstract | Title, Abstract |
| 2 | Scientific background | Introduction |
| 3 | Objectives | Introduction |
| 4 | Ecological cross-sectional design | §2.1 |
| 5 | Setting, aggregation level (country) | §2.1, §2.3.1 |
| 6 | Eligibility (countries with complete data) | §2.3.1 |
| 7 | Variables: outcome, exposures, confounders | §2.2, §2.4 |
| 8 | Data sources / measurement | §2.2 |
| 9 | Bias: ecological fallacy, detection bias | §4.5 |
| 10 | Study size (163 countries) | §2.3.1 |
| 12 | Statistical methods, confounder adjustment | §2.6–2.7 |
| 14 | Descriptive data | §3.1 |
| 16 | Main results with adjustment | §3.2, §3.8, Table 1 |
| 18 | Key results | §4.1 |
| 19 | Limitations (ecological, temporal, registry) | §4.5 |
| 22 | Funding | Funding statement |

**Supplementary Table S3. Sex-stratified association analysis**

*Spearman rank correlations of each dietary factor with thyroid cancer incidence ASR, computed separately for combined-sex, female and male rates (n = 163 countries).*

| **Dietary factor** | **Combined-sex ρ** | **Female ρ** | **Male ρ** |
| --- | --- | --- | --- |
| Iodine-rich food proxy | 0.234 | [from sex-specific extract] | [from sex-specific extract] |
| Total energy (kcal/capita/day) | 0.23 | [from sex-specific extract] | [from sex-specific extract] |
| Animal:plant protein ratio | 0.221 | [from sex-specific extract] | [from sex-specific extract] |
| Seafood supply (kg/capita/year) | 0.219 | [from sex-specific extract] | [from sex-specific extract] |
| Animal protein (g/capita/day) | 0.19 | [from sex-specific extract] | [from sex-specific extract] |
| Molluscs (kg/capita/year) | 0.187 | [from sex-specific extract] | [from sex-specific extract] |
| Western diet score | 0.181 | [from sex-specific extract] | [from sex-specific extract] |
| Dairy supply (kg/capita/year) | 0.176 | [from sex-specific extract] | [from sex-specific extract] |
| Obesity prevalence (%) | 0.17 | [from sex-specific extract] | [from sex-specific extract] |
| Mediterranean diet score | 0.141 | [from sex-specific extract] | [from sex-specific extract] |
| Total fat density | 0.066 | [from sex-specific extract] | [from sex-specific extract] |
| Plant protein (g/capita/day) | -0.167 | [from sex-specific extract] | [from sex-specific extract] |

**Supplementary Table S4. Multiple-imputation (MICE) sensitivity analysis**

*Change in mean, standard deviation and Spearman correlation before and after imputation for every imputed variable.*

| **Missing fraction** | **Variable** | **n missing** | **Δ mean** | **Δ SD** | **ρ(orig,imputed)** | **MSE** |
| --- | --- | --- | --- | --- | --- | --- |
| 0.05 | Iodine_rich_food_proxy | 10 | 0.045 | 0.009 | 0.999 | 3.96 |
| 0.05 | FAOSTAT_Seafood_Total | 13 | -0.001 | -0.002 | 1.0 | 0.003 |
| 0.05 | Animal_to_plant_protein_ratio | 9 | 0.001 | -0.001 | 0.999 | 0.004 |
| 0.05 | FAOSTAT_Milk_Excluding_Butter | 8 | 0.159 | 0.114 | 0.994 | 123.648 |
| 0.05 | Obesity_prevalence_pct | 8 | -0.002 | -0.001 | 1.0 | 0.031 |
| 0.1 | Iodine_rich_food_proxy | 14 | 0.011 | -0.026 | 0.999 | 3.116 |
| 0.1 | FAOSTAT_Seafood_Total | 11 | -0.005 | -0.015 | 1.0 | 0.214 |
| 0.1 | Animal_to_plant_protein_ratio | 25 | 0.003 | -0.011 | 0.966 | 0.028 |
| 0.1 | FAOSTAT_Milk_Excluding_Butter | 26 | 0.009 | -0.451 | 0.981 | 150.371 |
| 0.1 | Obesity_prevalence_pct | 14 | 0.021 | -0.043 | 0.999 | 0.59 |
| 0.2 | Iodine_rich_food_proxy | 39 | 0.237 | 0.02 | 0.99 | 9.983 |
| 0.2 | FAOSTAT_Seafood_Total | 45 | 0.03 | -0.015 | 0.999 | 0.591 |
| 0.2 | Animal_to_plant_protein_ratio | 27 | 0.002 | 0.003 | 0.989 | 0.006 |
| 0.2 | FAOSTAT_Milk_Excluding_Butter | 37 | 0.962 | -2.535 | 0.872 | 515.006 |
| 0.2 | Obesity_prevalence_pct | 30 | -0.026 | 0.014 | 0.995 | 1.171 |

**Supplementary Table S6. Unadjusted and confounder-adjusted associations with both multiplicity corrections**

| **Variable** | **ρ** | **q (FDR)** | **p (Bonf.)** | **Partial r (basic)** | **q (FDR)** | **p (Bonf.)** | **Partial r (extended)** | **q (FDR)** | **p (Bonf.)** |
| --- | --- | --- | --- | --- | --- | --- | --- | --- | --- |
| Iodine-rich food proxy | 0.234 | 0.016 | 0.034 | 0.231 | 0.019 | 0.038 | 0.224 | 0.026 | 0.052 |
| Total energy (kcal/capita/day) | 0.230 | 0.016 | 0.040 | 0.098 | 0.309 | 1.000 | 0.028 | 0.794 | 1.000 |
| Animal:plant protein ratio | 0.221 | 0.016 | 0.057 | 0.246 | 0.019 | 0.019 | 0.260 | 0.011 | 0.011 |
| Seafood supply (kg/capita/year) | 0.219 | 0.016 | 0.063 | 0.208 | 0.032 | 0.096 | 0.196 | 0.031 | 0.155 |
| Animal protein (g/capita/day) | 0.190 | 0.035 | 0.186 | 0.177 | 0.059 | 0.293 | 0.176 | 0.051 | 0.304 |
| Molluscs (kg/capita/year) | 0.187 | 0.035 | 0.209 | 0.184 | 0.059 | 0.234 | 0.203 | 0.031 | 0.120 |
| Western diet score | 0.181 | 0.037 | 0.260 | 0.043 | 0.710 | 1.000 | 0.040 | 0.739 | 1.000 |
| Dairy supply (kg/capita/year) | 0.176 | 0.038 | 0.304 | 0.158 | 0.089 | 0.536 | 0.198 | 0.031 | 0.141 |
| Obesity prevalence (%) | 0.170 | 0.042 | 0.377 | 0.020 | 0.872 | 1.000 | 0.157 | 0.079 | 0.556 |
| Mediterranean diet score | 0.141 | 0.080 | 0.883 | 0.145 | 0.113 | 0.792 | 0.127 | 0.145 | 1.000 |
| Total fat density | 0.066 | 0.406 | 1.000 | 0.007 | 0.925 | 1.000 | 0.008 | 0.918 | 1.000 |
| Plant protein (g/capita/day) | -0.167 | 0.042 | 0.417 | -0.095 | 0.309 | 1.000 | -0.149 | 0.088 | 0.705 |

*Note. Corrections applied within each family of 12 comparisons. The basic adjustment set contains HDI and obesity prevalence; the extended set adds the three HDI sub-indices and region fixed effects. The variable previously labelled “iodine-rich food proxy” is referred to throughout the revised manuscript as the iodine-rich food availability index; the variable itself is unchanged.*

**Supplementary Table S7. Feature-selection stability and Boruta confirmation**

| **Feature (by selection freq)** | **Sel. freq** | **Feature (by Boruta)** | **Boruta hit rate** |
| --- | --- | --- | --- |
| FAOSTAT_Wheat_and_products | 1.0 | HDI_standardized | 1.0 |
| FAOSTAT_Barley_and_products | 1.0 | HDI_squared | 1.0 |
| FAOSTAT_Maize_and_products | 1.0 | HDI_2023 | 1.0 |
| FAOSTAT_Pulses_Other | 1.0 | Income_index | 1.0 |
| FAOSTAT_Sugar_cane | 1.0 | Life_expectancy_index | 0.92 |
| PCA_component1_dietary | 1.0 | FAOSTAT_Dairy_energy | 0.65 |
| FAOSTAT_Animal_fat_density | 1.0 | FAOSTAT_Yams | 0.52 |
| FAOSTAT_Dairy_energy | 1.0 | Education_index | 0.52 |
| FAOSTAT_Cereals_energy | 1.0 | FAOSTAT_Offals_Edible | 0.42 |
| Life_expectancy_index | 1.0 | FAOSTAT_Sugar_cane | 0.35 |
| HDI_2023 | 1.0 | FAOSTAT_Egg_fat | 0.12 |
| Income_index | 1.0 | FAOSTAT_Poultry_Meat | 0.1 |
| Education_index | 1.0 | FAOSTAT_Mutton_and_Goat_Meat | 0.02 |
| FAOSTAT_Seafood_energy | 1.0 | FAOSTAT_Oranges_Mandarines | 0.02 |
| HDI_standardized | 1.0 | FAOSTAT_Plant_fat_density | 0.02 |

**Supplementary Table S8. Fold-level variability of regression performance under nested cross-validation**

| **Model** | **Pooled out-of-fold R²** | **95% bootstrap CI** | **SD of R² across 5 repetitions** | **RMSE** | **MAE** |
| --- | --- | --- | --- | --- | --- |
| Stacking Ensemble | 0.613 | [0.510, 0.726] | 0.015 | 3.04 | 1.82 |
| Random Forest | 0.609 | [0.501, 0.736] | 0.013 | 3.06 | 1.81 |
| Bagging | 0.599 | [0.485, 0.721] | 0.011 | 3.10 | 1.84 |
| KNN | 0.580 | [0.503, 0.672] | 0.042 | 3.17 | 2.01 |
| Lasso | 0.579 | [0.484, 0.690] | 0.023 | 3.17 | 2.01 |
| SVR (RBF) | 0.578 | [0.485, 0.689] | 0.010 | 3.18 | 1.96 |
| Extra Trees | 0.575 | [0.470, 0.697] | 0.011 | 3.19 | 1.85 |
| ElasticNet | 0.571 | [0.490, 0.669] | 0.012 | 3.20 | 2.10 |
| Ridge | 0.562 | [0.477, 0.656] | 0.012 | 3.23 | 2.19 |
| Bayesian Ridge | 0.561 | [0.476, 0.657] | 0.014 | 3.24 | 2.20 |
| MLP | 0.522 | [0.409, 0.612] | 0.027 | 3.38 | 2.31 |
| Gradient Boosting | 0.448 | [0.232, 0.624] | 0.060 | 3.63 | 2.11 |
| Linear Regression | 0.312 | [-0.146, 0.564] | 0.037 | 4.05 | 2.58 |

**Supplementary Table S9. Sensitivity analysis — models fitted to all 150 features with no feature-selection step**

| **Model** | **Pooled out-of-fold R² (no selection)** | **R² with nested consensus selection** | **Difference** | **Spearman ρ (no selection)** |
| --- | --- | --- | --- | --- |
| Extra Trees | 0.562 | 0.575 | -0.013 | 0.900 |
| Random Forest | 0.622 | 0.609 | +0.013 | 0.897 |
| Bayesian Ridge | 0.544 | 0.561 | -0.017 | 0.850 |
| Gradient Boosting | 0.495 | 0.448 | +0.048 | 0.882 |

**Supplementary Table S10. Block-wise models and the incremental predictive value of dietary features**

| **Model** | **Feature block** | **Variables** | **Pooled out-of-fold R²** | **95% bootstrap CI** | **RMSE** | **Spearman ρ** |
| --- | --- | --- | --- | --- | --- | --- |
| Bayesian Ridge | Socioeconomic only | 9 | 0.609 | [0.487, 0.760] | 3.06 | 0.902 |
| Bayesian Ridge | Dietary only | 140 | 0.284 | [0.125, 0.387] | 4.14 | 0.450 |
| Bayesian Ridge | Combined | 150 | 0.561 | [0.476, 0.657] | 3.24 | 0.887 |
| Random Forest | Socioeconomic only | 9 | 0.589 | [0.460, 0.740] | 3.14 | 0.899 |
| Random Forest | Dietary only | 140 | 0.257 | [0.112, 0.356] | 4.21 | 0.421 |
| Random Forest | Combined | 150 | 0.609 | [0.501, 0.736] | 3.06 | 0.896 |
| Extra Trees | Socioeconomic only | 9 | 0.558 | [0.399, 0.723] | 3.25 | 0.899 |
| Extra Trees | Dietary only | 140 | 0.228 | [0.061, 0.327] | 4.30 | 0.430 |
| Extra Trees | Combined | 150 | 0.575 | [0.470, 0.697] | 3.19 | 0.904 |

**S10b. Incremental value of the dietary block over the socioeconomic block**

| **Model** | **ΔR² (combined − socioeconomic only)** | **95% paired bootstrap CI** | **Bootstrap p** |
| --- | --- | --- | --- |
| Bayesian Ridge | -0.048 | [-0.169, +0.034] | 0.266 |
| Random Forest | +0.021 | [-0.059, +0.079] | 0.579 |
| Extra Trees | +0.017 | [-0.084, +0.133] | 0.754 |

**Supplementary Table S11. Leave-one-region-out validation**

| **Model** | **Held-out region** | **n** | **Held-out R²** | **RMSE** | **Spearman ρ** |
| --- | --- | --- | --- | --- | --- |
| Bayesian Ridge | Africa | 51 | -2.785 | 2.43 | 0.777 |
| Bayesian Ridge | Asia | 43 | 0.324 | 4.89 | 0.766 |
| Bayesian Ridge | Europe | 34 | -1.516 | 3.50 | 0.402 |
| Bayesian Ridge | Northern America & Caribbean | 14 | 0.441 | 2.76 | 0.679 |
| Bayesian Ridge | Oceania | 11 | 0.601 | 3.20 | 0.864 |
| Bayesian Ridge | South America | 10 | -139.734 | 4.04 | 0.333 |
| Bayesian Ridge | POOLED (all regions held out) | 163 | 0.453 | 3.61 | 0.850 |
| Random Forest | Africa | 51 | -0.684 | 1.62 | 0.582 |
| Random Forest | Asia | 43 | 0.448 | 4.42 | 0.840 |
| Random Forest | Europe | 34 | -1.212 | 3.28 | 0.525 |
| Random Forest | Northern America & Caribbean | 14 | 0.598 | 2.34 | 0.789 |
| Random Forest | Oceania | 11 | 0.312 | 4.20 | 0.691 |
| Random Forest | South America | 10 | -95.811 | 3.35 | 0.430 |
| Random Forest | POOLED (all regions held out) | 163 | 0.558 | 3.25 | 0.813 |

**Supplementary Table S12. Classification sensitivity analysis at two thresholds**

*This table was presented as Table 3 in the previous version of the manuscript and is relocated here because the classification analysis is a sensitivity analysis on which no conclusion of the study depends.*

| **Threshold** | **Model** | **AUC-ROC** | **95% bootstrap CI** | **AUPRC** | **Brier** | **Accuracy** | **F1** |
| --- | --- | --- | --- | --- | --- | --- | --- |
| Median split | Extra Trees | 0.932 | [0.893, 0.964] | 0.933 | 0.110 | 0.847 | 0.848 |
| Median split | Random Forest | 0.928 | [0.888, 0.961] | 0.926 | 0.110 | 0.840 | 0.845 |
| Median split | SVM (RBF) | 0.920 | [0.874, 0.959] | 0.906 | 0.110 | 0.840 | 0.845 |
| Median split | Gradient Boosting | 0.917 | [0.872, 0.955] | 0.909 | 0.125 | 0.834 | 0.836 |
| Median split | Logistic Regression | 0.915 | [0.869, 0.955] | 0.918 | 0.121 | 0.840 | 0.843 |
| Median split | KNN | 0.903 | [0.853, 0.945] | 0.893 | 0.124 | 0.847 | 0.847 |
| Median split | MLP | 0.882 | [0.826, 0.933] | 0.876 | 0.162 | 0.810 | 0.810 |
| Upper quartile | Random Forest | 0.985 | [0.970, 0.996] | 0.960 | 0.045 | 0.945 | 0.889 |
| Upper quartile | Extra Trees | 0.984 | [0.967, 0.996] | 0.960 | 0.049 | 0.945 | 0.886 |
| Upper quartile | SVM (RBF) | 0.967 | [0.938, 0.989] | 0.906 | 0.066 | 0.908 | 0.805 |
| Upper quartile | Logistic Regression | 0.965 | [0.934, 0.987] | 0.915 | 0.064 | 0.933 | 0.861 |
| Upper quartile | KNN | 0.940 | [0.905, 0.970] | 0.837 | 0.092 | 0.847 | 0.615 |
| Upper quartile | Gradient Boosting | 0.909 | [0.837, 0.970] | 0.815 | 0.079 | 0.914 | 0.825 |
| Upper quartile | MLP | 0.852 | [0.783, 0.912] | 0.719 | 0.148 | 0.828 | 0.517 |

**Supplementary Table S5. Complete variable dictionary (150 numeric predictors)**

| **Variable** | **Source** | **Year** | **Unit** | **Missingness** | **Imputation** | **Lag** | **Selection freq** |
| --- | --- | --- | --- | --- | --- | --- | --- |
| FAOSTAT_Wheat_and_products | FAOSTAT FBS | 2015–2019 mean | kg/capita/year | 0.0% | nan | — | 1.0 |
| FAOSTAT_Rice_Milled_Equivalent | FAOSTAT FBS | 2015–2019 mean | kg/capita/year | 0.0% | nan | — | 0.0 |
| FAOSTAT_Barley_and_products | FAOSTAT FBS | 2015–2019 mean | kg/capita/year | 0.0% | nan | — | 1.0 |
| FAOSTAT_Maize_and_products | FAOSTAT FBS | 2015–2019 mean | kg/capita/year | 0.0% | nan | — | 1.0 |
| FAOSTAT_Rye_and_products | FAOSTAT FBS | 2015–2019 mean | kg/capita/year | 0.0% | nan | — | 0.68 |
| FAOSTAT_Oats | FAOSTAT FBS | 2015–2019 mean | kg/capita/year | 0.0% | nan | — | 0.16 |
| FAOSTAT_Millet_and_products | FAOSTAT FBS | 2015–2019 mean | kg/capita/year | 0.0% | nan | — | 0.0 |
| FAOSTAT_Sorghum_and_products | FAOSTAT FBS | 2015–2019 mean | kg/capita/year | 0.0% | nan | — | 0.68 |
| FAOSTAT_Cereals_Other | FAOSTAT FBS | 2015–2019 mean | kg/capita/year | 0.0% | nan | — | 0.0 |
| FAOSTAT_Cassava_and_products | FAOSTAT FBS | 2015–2019 mean | kg/capita/year | 0.0% | nan | — | 0.16 |
| FAOSTAT_Potatoes_and_products | FAOSTAT FBS | 2015–2019 mean | kg/capita/year | 0.0% | nan | — | 0.0 |
| FAOSTAT_Sweet_potatoes | FAOSTAT FBS | 2015–2019 mean | kg/capita/year | 0.0% | nan | — | 0.0 |
| FAOSTAT_Yams | FAOSTAT FBS | 2015–2019 mean | kg/capita/year | 0.0% | nan | — | 0.94 |
| FAOSTAT_Roots_and_Tubers_Other | FAOSTAT FBS | 2015–2019 mean | kg/capita/year | 0.0% | nan | — | 0.0 |
| FAOSTAT_Sugar_cane | FAOSTAT FBS | 2015–2019 mean | kg/capita/year | 0.0% | nan | — | 1.0 |
| FAOSTAT_Sugar_beet | FAOSTAT FBS | 2015–2019 mean | kg/capita/year | 0.0% | nan | — | 0.08 |
| FAOSTAT_Sugar_and_Sweeteners | FAOSTAT FBS | 2015–2019 mean | kg/capita/year | 0.0% | nan | — | 0.36 |
| FAOSTAT_Beans | FAOSTAT FBS | 2015–2019 mean | kg/capita/year | 0.0% | nan | — | 0.96 |
| FAOSTAT_Peas | FAOSTAT FBS | 2015–2019 mean | kg/capita/year | 0.0% | nan | — | 0.0 |
| FAOSTAT_Pulses_Other | FAOSTAT FBS | 2015–2019 mean | kg/capita/year | 0.0% | nan | — | 1.0 |
| FAOSTAT_Tomatoes_and_products | FAOSTAT FBS | 2015–2019 mean | kg/capita/year | 0.0% | nan | — | 0.74 |
| FAOSTAT_Onions | FAOSTAT FBS | 2015–2019 mean | kg/capita/year | 0.0% | nan | — | 0.06 |
| FAOSTAT_Vegetables_Other | FAOSTAT FBS | 2015–2019 mean | kg/capita/year | 0.0% | nan | — | 0.14 |
| FAOSTAT_Oranges_Mandarines | FAOSTAT FBS | 2015–2019 mean | kg/capita/year | 0.0% | nan | — | 1.0 |
| FAOSTAT_Bananas | FAOSTAT FBS | 2015–2019 mean | kg/capita/year | 0.0% | nan | — | 0.98 |
| FAOSTAT_Apples_and_products | FAOSTAT FBS | 2015–2019 mean | kg/capita/year | 0.0% | nan | — | 0.22 |
| FAOSTAT_Fruits_Other | FAOSTAT FBS | 2015–2019 mean | kg/capita/year | 0.0% | nan | — | 0.28 |
| FAOSTAT_Treenuts | FAOSTAT FBS | 2015–2019 mean | kg/capita/year | 0.0% | nan | — | 0.0 |
| FAOSTAT_Groundnuts_Shelled_Equivalent | FAOSTAT FBS | 2015–2019 mean | kg/capita/year | 0.0% | nan | — | 0.08 |
| FAOSTAT_Soybeans | FAOSTAT FBS | 2015–2019 mean | kg/capita/year | 0.0% | nan | — | 0.92 |
| FAOSTAT_Rape_and_Mustard_Oil | FAOSTAT FBS | 2015–2019 mean | kg/capita/year | 0.0% | nan | — | 0.0 |
| FAOSTAT_Palm_Oil | FAOSTAT FBS | 2015–2019 mean | kg/capita/year | 0.0% | nan | — | 0.0 |
| FAOSTAT_Sunflower_Oil | FAOSTAT FBS | 2015–2019 mean | kg/capita/year | 0.0% | nan | — | 0.08 |
| FAOSTAT_Olive_Oil | FAOSTAT FBS | 2015–2019 mean | kg/capita/year | 0.0% | nan | — | 0.0 |
| FAOSTAT_Vegetable_Oils_Other | FAOSTAT FBS | 2015–2019 mean | kg/capita/year | 0.0% | nan | — | 1.0 |
| FAOSTAT_Bovine_Meat | FAOSTAT FBS | 2015–2019 mean | kg/capita/year | 0.0% | nan | — | 0.0 |
| FAOSTAT_Mutton_and_Goat_Meat | FAOSTAT FBS | 2015–2019 mean | kg/capita/year | 0.0% | nan | — | 0.9 |
| FAOSTAT_Pig_Meat | FAOSTAT FBS | 2015–2019 mean | kg/capita/year | 0.0% | nan | — | 0.02 |
| FAOSTAT_Poultry_Meat | FAOSTAT FBS | 2015–2019 mean | kg/capita/year | 0.0% | nan | — | 0.96 |
| FAOSTAT_Horse_Meat | FAOSTAT FBS | 2015–2019 mean | kg/capita/year | 0.0% | nan | — | 0.38 |
| FAOSTAT_Offals_Edible | FAOSTAT FBS | 2015–2019 mean | kg/capita/year | 0.0% | nan | — | 1.0 |
| FAOSTAT_Meat_Other | FAOSTAT FBS | 2015–2019 mean | kg/capita/year | 0.0% | nan | — | 0.0 |
| FAOSTAT_Animal_Products | FAOSTAT FBS | 2015–2019 mean | kg/capita/year | 0.0% | nan | — | 0.5 |
| FAOSTAT_Animal_fats | FAOSTAT FBS | 2015–2019 mean | kg/capita/year | 0.0% | nan | — | 1.0 |
| FAOSTAT_Butter_Ghee | FAOSTAT FBS | 2015–2019 mean | kg/capita/year | 0.0% | nan | — | 0.26 |
| FAOSTAT_Cream | FAOSTAT FBS | 2015–2019 mean | kg/capita/year | 0.0% | nan | — | 0.02 |
| FAOSTAT_Milk_Excluding_Butter | FAOSTAT FBS | 2015–2019 mean | kg/capita/year | 0.0% | nan | — | 0.68 |
| FAOSTAT_Cheese | FAOSTAT FBS | 2015–2019 mean | kg/capita/year | 0.0% | nan | — | 0.84 |
| FAOSTAT_Eggs | FAOSTAT FBS | 2015–2019 mean | kg/capita/year | 0.0% | nan | — | 0.46 |
| FAOSTAT_Fish_Freshwater | FAOSTAT FBS | 2015–2019 mean | kg/capita/year | 0.0% | nan | — | 0.0 |
| FAOSTAT_Fish_Demersal | FAOSTAT FBS | 2015–2019 mean | kg/capita/year | 0.0% | nan | — | 0.16 |
| FAOSTAT_Fish_Pelagic | FAOSTAT FBS | 2015–2019 mean | kg/capita/year | 0.0% | nan | — | 0.0 |
| FAOSTAT_Fish_Marine_Other | FAOSTAT FBS | 2015–2019 mean | kg/capita/year | 0.0% | nan | — | 0.0 |
| FAOSTAT_Crustaceans | FAOSTAT FBS | 2015–2019 mean | kg/capita/year | 0.0% | nan | — | 0.0 |
| FAOSTAT_Cephalopods | FAOSTAT FBS | 2015–2019 mean | kg/capita/year | 0.0% | nan | — | 0.9 |
| FAOSTAT_Molluscs_excl_Cephalopods | FAOSTAT FBS | 2015–2019 mean | kg/capita/year | 0.0% | nan | — | 0.04 |
| FAOSTAT_Aquatic_Animals_Other | FAOSTAT FBS | 2015–2019 mean | kg/capita/year | 0.0% | nan | — | 1.0 |
| FAOSTAT_Fish_Preserved | FAOSTAT FBS | 2015–2019 mean | kg/capita/year | 0.0% | nan | — | 0.92 |
| FAOSTAT_Fish_Body_Oil | FAOSTAT FBS | 2015–2019 mean | kg/capita/year | 0.0% | nan | — | 0.28 |
| FAOSTAT_Fish_Liver_Oil | FAOSTAT FBS | 2015–2019 mean | kg/capita/year | 0.0% | nan | — | 0.78 |
| FAOSTAT_Aquatic_Plants | FAOSTAT FBS | 2015–2019 mean | kg/capita/year | 0.0% | nan | — | 0.0 |
| FAOSTAT_Seafood_Total | FAOSTAT FBS | 2015–2019 mean | kg/capita/year | 0.0% | nan | — | 0.36 |
| FAOSTAT_Wine | FAOSTAT FBS | 2015–2019 mean | kg/capita/year | 0.0% | nan | — | 0.48 |
| FAOSTAT_Beer | FAOSTAT FBS | 2015–2019 mean | kg/capita/year | 0.0% | nan | — | 0.0 |
| FAOSTAT_Beverages_Alcoholic | FAOSTAT FBS | 2015–2019 mean | kg/capita/year | 0.0% | nan | — | 0.06 |
| FAOSTAT_Coffee_and_products | FAOSTAT FBS | 2015–2019 mean | kg/capita/year | 0.0% | nan | — | 0.22 |
| FAOSTAT_Tea_including_mate | FAOSTAT FBS | 2015–2019 mean | kg/capita/year | 0.0% | nan | — | 0.1 |
| FAOSTAT_Cocoa_Beans_and_products | FAOSTAT FBS | 2015–2019 mean | kg/capita/year | 0.0% | nan | — | 0.04 |
| FAOSTAT_Pepper | FAOSTAT FBS | 2015–2019 mean | kg/capita/year | 0.0% | nan | — | 0.36 |
| FAOSTAT_Spices_Other | FAOSTAT FBS | 2015–2019 mean | kg/capita/year | 0.0% | nan | — | 0.0 |
| FAOSTAT_Honey | FAOSTAT FBS | 2015–2019 mean | kg/capita/year | 0.0% | nan | — | 1.0 |
| FAOSTAT_Infant_food | FAOSTAT FBS | 2015–2019 mean | kg/capita/year | 0.0% | nan | — | 0.0 |
| FAOSTAT_Miscellaneous | FAOSTAT FBS | 2015–2019 mean | kg/capita/year | 0.0% | nan | — | 0.02 |
| FAOSTAT_Total_calories_kcal/day | FAOSTAT FBS | 2015–2019 mean | kcal/capita/day | 0.0% | nan | — | 0.16 |
| FAOSTAT_Total_protein_g/day | FAOSTAT FBS | 2015–2019 mean | kg/capita/year | 0.0% | nan | — | 0.0 |
| FAOSTAT_Total_fat_g/day | FAOSTAT FBS | 2015–2019 mean | kg/capita/year | 0.0% | nan | — | 0.02 |
| FAOSTAT_Wheat_protein | FAOSTAT FBS | 2015–2019 mean | g/capita/day | 0.0% | nan | — | 0.06 |
| FAOSTAT_Rice_protein | FAOSTAT FBS | 2015–2019 mean | g/capita/day | 0.0% | nan | — | 0.98 |
| FAOSTAT_Maize_protein | FAOSTAT FBS | 2015–2019 mean | g/capita/day | 0.0% | nan | — | 1.0 |
| FAOSTAT_Meat_protein | FAOSTAT FBS | 2015–2019 mean | g/capita/day | 0.0% | nan | — | 0.08 |
| FAOSTAT_Dairy_protein | FAOSTAT FBS | 2015–2019 mean | g/capita/day | 0.0% | nan | — | 0.02 |
| FAOSTAT_Fish_protein | FAOSTAT FBS | 2015–2019 mean | g/capita/day | 0.0% | nan | — | 0.8 |
| FAOSTAT_Legume_protein | FAOSTAT FBS | 2015–2019 mean | g/capita/day | 0.0% | nan | — | 0.08 |
| FAOSTAT_Plant_protein_total | FAOSTAT FBS | 2015–2019 mean | g/capita/day | 0.0% | nan | — | 0.0 |
| FAOSTAT_Animal_protein_total | FAOSTAT FBS | 2015–2019 mean | g/capita/day | 0.0% | nan | — | 0.22 |
| FAOSTAT_Wheat_fat | FAOSTAT FBS | 2015–2019 mean | g/capita/day | 0.0% | nan | — | 0.62 |
| FAOSTAT_Rice_fat | FAOSTAT FBS | 2015–2019 mean | g/capita/day | 0.0% | nan | — | 0.44 |
| FAOSTAT_Meat_fat | FAOSTAT FBS | 2015–2019 mean | g/capita/day | 0.0% | nan | — | 0.7 |
| FAOSTAT_Dairy_fat | FAOSTAT FBS | 2015–2019 mean | g/capita/day | 0.0% | nan | — | 0.0 |
| FAOSTAT_Fish_fat | FAOSTAT FBS | 2015–2019 mean | g/capita/day | 0.0% | nan | — | 0.52 |
| FAOSTAT_Oil_fat | FAOSTAT FBS | 2015–2019 mean | g/capita/day | 0.0% | nan | — | 0.04 |
| FAOSTAT_Total_fat_density | FAOSTAT FBS | 2015–2019 mean | g/1000 kcal | 0.0% | nan | — | 0.0 |
| FAOSTAT_Animal_fat_density | FAOSTAT FBS | 2015–2019 mean | g/1000 kcal | 0.0% | nan | — | 1.0 |
| FAOSTAT_Plant_fat_density | FAOSTAT FBS | 2015–2019 mean | g/1000 kcal | 0.0% | nan | — | 0.24 |
| FAOSTAT_Seafood_protein | FAOSTAT FBS | 2015–2019 mean | g/capita/day | 0.0% | nan | — | 0.9 |
| FAOSTAT_Molluscs_protein | FAOSTAT FBS | 2015–2019 mean | g/capita/day | 0.0% | nan | — | 0.94 |
| FAOSTAT_Crustaceans_protein | FAOSTAT FBS | 2015–2019 mean | g/capita/day | 0.0% | nan | — | 0.0 |
| FAOSTAT_Dairy_energy | FAOSTAT FBS | 2015–2019 mean | kcal/capita/day | 0.0% | nan | — | 1.0 |
| FAOSTAT_Meat_energy | FAOSTAT FBS | 2015–2019 mean | kcal/capita/day | 0.0% | nan | — | 0.0 |
| FAOSTAT_Cereals_energy | FAOSTAT FBS | 2015–2019 mean | kcal/capita/day | 0.0% | nan | — | 1.0 |
| FAOSTAT_Fruits_energy | FAOSTAT FBS | 2015–2019 mean | kcal/capita/day | 0.0% | nan | — | 0.06 |
| FAOSTAT_Vegetables_energy | FAOSTAT FBS | 2015–2019 mean | kcal/capita/day | 0.0% | nan | — | 0.48 |
| FAOSTAT_Seafood_energy | FAOSTAT FBS | 2015–2019 mean | kcal/capita/day | 0.0% | nan | — | 1.0 |
| FAOSTAT_Sugar_energy | FAOSTAT FBS | 2015–2019 mean | kcal/capita/day | 0.0% | nan | — | 0.72 |
| FAOSTAT_Oil_energy | FAOSTAT FBS | 2015–2019 mean | kcal/capita/day | 0.0% | nan | — | 0.56 |
| FAOSTAT_Pulses_energy | FAOSTAT FBS | 2015–2019 mean | kcal/capita/day | 0.0% | nan | — | 0.6 |
| FAOSTAT_Egg_protein | FAOSTAT FBS | 2015–2019 mean | g/capita/day | 0.0% | nan | — | 0.02 |
| FAOSTAT_Egg_fat | FAOSTAT FBS | 2015–2019 mean | g/capita/day | 0.0% | nan | — | 0.0 |
| FAOSTAT_Bovine_fat | FAOSTAT FBS | 2015–2019 mean | g/capita/day | 0.0% | nan | — | 0.0 |
| FAOSTAT_Poultry_protein | FAOSTAT FBS | 2015–2019 mean | g/capita/day | 0.0% | nan | — | 0.72 |
| FAOSTAT_Lamb_and_Mutton_fat | FAOSTAT FBS | 2015–2019 mean | g/capita/day | 0.0% | nan | — | 0.0 |
| FAOSTAT_Pork_fat | FAOSTAT FBS | 2015–2019 mean | g/capita/day | 0.0% | nan | — | 0.0 |
| FAOSTAT_Nuts_fat | FAOSTAT FBS | 2015–2019 mean | g/capita/day | 0.0% | nan | — | 0.94 |
| HDI_2023 | UNDP HDR 2025 | 2023 | index 0–1 | 1.84% | MICE within folds | — | 1.0 |
| Obesity_prevalence_pct | WHO GHO | 2022 | % adults BMI≥30 | 0.0% | nan | — | 0.06 |
| Life_expectancy_index | UNDP HDR 2025 | 2023 | index 0–1 | 1.84% | MICE within folds | — | 1.0 |
| Education_index | UNDP HDR 2025 | 2023 | index 0–1 | 1.84% | MICE within folds | — | 1.0 |
| Income_index | UNDP HDR 2025 | 2023 | index 0–1 | 1.84% | MICE within folds | — | 1.0 |
| Iodine_rich_food_proxy | Derived | — | composite score | 0.0% | nan | — | 0.98 |
| Western_diet_score | Derived | — | composite score | 0.0% | nan | — | 0.02 |
| Mediterranean_diet_score | Derived | — | composite score | 0.0% | nan | — | 0.04 |
| Dietary_diversity_index | Derived | — | composite score | 0.0% | nan | — | 0.0 |
| Animal_to_plant_protein_ratio | Derived | — | — | 0.0% | nan | — | 0.26 |
| Fat_density | Derived | — | — | 0.0% | nan | — | 0.0 |
| UPF_proxy_exploratory | Derived | — | composite score | 0.0% | nan | — | 0.84 |
| Seafood_diversity_score | Derived | — | composite score | 0.0% | nan | — | 0.0 |
| Lag0_seafood_supply | FAOSTAT FBS | ending 2022 | kg/capita/year | 0.0% | nan | 0 yr window | 0.44 |
| Lag3yr_seafood_supply | FAOSTAT FBS | ending 2019 | kg/capita/year | 0.0% | nan | 3 yr window | 0.64 |
| Lag5yr_seafood_supply | FAOSTAT FBS | ending 2017 | kg/capita/year | 0.0% | nan | 5 yr window | 0.38 |
| Lag10yr_seafood_supply | FAOSTAT FBS | ending 2012 | kg/capita/year | 0.0% | nan | 10 yr window | 0.48 |
| Lag12yr_seafood_supply | FAOSTAT FBS | ending 2010 | kg/capita/year | 0.0% | nan | 12 yr window | 0.58 |
| Dietary_cluster_KMeans4 | Derived | — | — | 0.0% | nan | — | 0.0 |
| PCA_component1_dietary | Derived | — | component score | 0.0% | nan | — | 1.0 |
| PCA_component2_dietary | Derived | — | component score | 0.0% | nan | — | 0.0 |
| Seafood_x_HDI_interaction | Derived | — | standardized product | 1.84% | MICE within folds | — | 0.0 |
| Seafood_per_capita_std | Derived | — | — | 0.0% | nan | — | 0.4 |
| HDI_standardized | UNDP HDR 2025 | 2023 | index 0–1 | 1.84% | MICE within folds | — | 1.0 |
| Obesity_standardized | WHO GHO | 2022 | z-score | 0.0% | nan | — | 0.06 |
| Animal_protein_density | Derived | — | — | 0.0% | nan | — | 0.44 |
| Plant_protein_density | Derived | — | — | 0.0% | nan | — | 0.5 |
| Molluscs_log1p | Derived | — | — | 0.0% | nan | — | 0.02 |
| Seafood_log1p | Derived | — | — | 0.0% | nan | — | 0.16 |
| Total_fat_log1p | Derived | — | — | 0.0% | nan | — | 0.0 |
| Animal_products_log1p | Derived | — | — | 0.0% | nan | — | 0.52 |
| Iodine_proxy_log1p | Derived | — | — | 0.0% | nan | — | 0.86 |
| HDI_squared | UNDP HDR 2025 | 2023 | index 0–1 | 1.84% | MICE within folds | — | 1.0 |
| Obesity_x_HDI | Derived | — | standardized product | 1.84% | MICE within folds | — | 0.04 |
| Seafood_x_obesity | Derived | — | standardized product | 0.0% | nan | — | 1.0 |
| High_iodine_flag | Derived | — | — | 0.0% | nan | — | 0.1 |
| High_plant_protein_flag | Derived | — | — | 0.0% | nan | — | 0.0 |

**Supplementary Table S13. Individual components of the iodine-rich food availability index, entered separately**

*Seafood supply, dairy supply (milk excluding butter) and egg supply were each entered as the exposure in place of the composite index, under the same three adjustment sets and the same 161 countries as Table 2 of the main text. Benjamini–Hochberg correction was applied within this family of four comparisons.*

| **Variable** | **Unadjusted ρ** | **q (FDR)** | **Partial r (basic)** | **q (FDR)** | **Partial r (extended)** | **q (FDR)** | **Partial r (ext. + animal protein)** | **q (FDR)** |
| --- | --- | --- | --- | --- | --- | --- | --- | --- |
| Seafood supply (kg/capita/year) | 0.219 | 0.008 | 0.208 | 0.016 | 0.196 | 0.017 | 0.176 | 0.034 |
| Dairy (milk excl. butter, kg/capita/year) | 0.176 | 0.025 | 0.158 | 0.054 | 0.198 | 0.017 | 0.190 | 0.032 |
| Egg supply (kg/capita/year) | 0.217 | 0.008 | 0.152 | 0.054 | 0.065 | 0.411 | 0.024 | 0.762 |
| Iodine-rich food availability index | 0.234 | 0.008 | 0.231 | 0.013 | 0.224 | 0.017 | 0.203 | 0.032 |

**Supplementary Table S14. Joint multivariable model containing all three components simultaneously**

*Standardised regression coefficients (change in incidence ASR per 1 SD of supply) from a single model containing seafood, dairy and egg supply together with the confounder set. Variance inflation factors were 1.07 (seafood), 1.03 (dairy) and 1.05 (eggs), and pairwise Spearman correlations among the three components were 0.045–0.184, indicating that the components are close to orthogonal.*

| **Adjustment set** | **Term** | **β (per SD)** | **SE** | **t** | **p** | **95% CI** |
| --- | --- | --- | --- | --- | --- | --- |
| Basic adjustment (HDI, obesity) | Seafood (z) | 0.537 | 0.252 | 2.14 | 0.034 | [0.044, 1.030] |
| Basic adjustment (HDI, obesity) | Dairy (z) | 0.418 | 0.247 | 1.69 | 0.092 | [-0.066, 0.901] |
| Basic adjustment (HDI, obesity) | Eggs (z) | 0.376 | 0.250 | 1.50 | 0.135 | [-0.115, 0.867] |
| Extended adjustment (+HDI sub-indices, region) | Seafood (z) | 0.476 | 0.243 | 1.96 | 0.052 | [-0.001, 0.953] |
| Extended adjustment (+HDI sub-indices, region) | Dairy (z) | 0.478 | 0.234 | 2.04 | 0.043 | [0.020, 0.937] |
| Extended adjustment (+HDI sub-indices, region) | Eggs (z) | 0.106 | 0.239 | 0.44 | 0.657 | [-0.362, 0.574] |

**Supplementary Table S15. Alternative weighting schemes for the iodine-rich food availability index**

*The composite was rebuilt under three alternative weighting schemes and compared with the primary construction (seafood + 0.10 × dairy + 0.05 × eggs) and with a seafood-only variant. Iodine-content weights are the ratios of published mean iodine concentrations for the three food groups (approximately 300, 150 and 250 μg/kg fresh weight; Zimmermann, 2023). Benjamini–Hochberg correction was applied within this family of five comparisons. Pairwise Spearman correlations among the five variants ranged from 0.56 to 0.98.*

| **Variable** | **Unadjusted ρ** | **q (FDR)** | **Partial r (basic)** | **q (FDR)** | **Partial r (extended)** | **q (FDR)** | **Partial r (ext. + animal protein)** | **q (FDR)** |
| --- | --- | --- | --- | --- | --- | --- | --- | --- |
| Primary index (1.00 / 0.10 / 0.05) | 0.234 | 0.003 | 0.231 | 0.004 | 0.224 | 0.005 | 0.203 | 0.012 |
| Equal-weighted sum (1 / 1 / 1) | 0.279 | 0.001 | 0.246 | 0.003 | 0.250 | 0.003 | 0.229 | 0.009 |
| Equal-weighted z-score sum | 0.293 | 0.000 | 0.270 | 0.002 | 0.238 | 0.004 | 0.206 | 0.012 |
| Iodine-content-weighted (1 / 0.50 / 0.83) | 0.291 | 0.000 | 0.267 | 0.002 | 0.256 | 0.003 | 0.231 | 0.009 |
| Seafood-only (1 / 0 / 0) | 0.219 | 0.005 | 0.208 | 0.008 | 0.196 | 0.013 | 0.176 | 0.025 |

**Supplementary Table S16. Specificity: iodine-rich versus iodine-poor animal-origin food availability**

*The iodine-poor animal-origin food index is the sum of per-capita supply of bovine meat, pig meat, poultry meat, and mutton and goat meat — the FAOSTAT animal-origin categories that are poor dietary sources of iodine. If the association of the iodine-rich food availability index reflected animal-origin food availability in general, this control index would be expected to behave similarly under adjustment. It does not. Spearman correlation between the iodine-rich food availability index and total animal protein supply was 0.109, and between the index and the iodine-poor index 0.165. Benjamini–Hochberg correction was applied within this family of four comparisons.*

| **Variable** | **Unadjusted ρ** | **q (FDR)** | **Partial r (basic)** | **q (FDR)** | **Partial r (extended)** | **q (FDR)** | **Partial r (ext. + animal protein)** | **q (FDR)** |
| --- | --- | --- | --- | --- | --- | --- | --- | --- |
| Iodine-rich food availability index | 0.234 | 0.006 | 0.231 | 0.013 | 0.224 | 0.017 | 0.203 | 0.039 |
| Iodine-poor animal foods (meat + poultry) | 0.308 | 0.000 | 0.135 | 0.087 | 0.102 | 0.198 | 0.088 | 0.537 |
| Total animal protein supply | 0.190 | 0.016 | 0.177 | 0.033 | 0.176 | 0.035 | -0.004 | 0.961 |
| Total animal products supply | 0.225 | 0.006 | 0.190 | 0.032 | 0.175 | 0.035 | 0.024 | 0.961 |

**Supplementary Figures**

*Supplementary Figures S1–S15 present the secondary and exploratory analyses relocated from the main text. Supplementary Figure S16 is new.*

Supplementary Figure S1. Distribution of thyroid cancer outcome variables across 163 countries. (A) Incidence ASR histogram with Shapiro–Wilk test (W = 0.83, p = 1.20e-12) and median. (B) Mortality ASR histogram. (C) Mortality-to-incidence ratio histogram. (D) Log₁₀-transformed incidence ASR, showing improved symmetry (W = 0.95, p = 1.27e-05). (E) Normal Q–Q plot of log-transformed incidence. (F) Incidence versus mortality ASR coloured by HDI. Incidence in high-HDI countries is disproportionately high relative to mortality, the pattern expected when detection intensity rather than disease occurrence drives differences between countries.

Supplementary Figure S2. Nine dietary and metabolic factors plotted against thyroid cancer incidence ASR across the 161 countries with complete socioeconomic data, each with a quadratic fit (red) and its Spearman ρ. Significance markers refer to FDR-corrected unadjusted p-values (*** q < 0.001, ** q < 0.01, * q < 0.05, ns = not significant). Unadjusted associations were positive and significant after FDR correction for the iodine-rich food availability index (ρ = 0.234, q = 0.016), total energy (ρ = 0.230, q = 0.016), the animal-to-plant protein ratio (ρ = 0.221, q = 0.016) and seafood supply (ρ = 0.219, q = 0.016), while plant protein was inversely associated (ρ = -0.167, q = 0.042). Table 2 and Figure 7 of the main text report which of these associations persist after confounder adjustment.

Supplementary Figure S3. HDI-stratified box plots with jittered individual observations for six cancer and dietary variables. Each panel reports the Kruskal–Wallis statistic and p-value, and group sizes are shown on the horizontal axis. Group boundaries are the official UNDP human development category thresholds (0.55, 0.70, 0.80); the groups are therefore of unequal size and are not quartiles. Incidence rose monotonically across the four categories and every dietary variable differed significantly between groups, but the within-group spread of incidence was widest in the very high category, so development level alone does not account for the heterogeneity observed among wealthy countries — a pattern equally consistent with differences in screening policy as with differences in diet.

Supplementary Figure S4. Principal component analysis of national dietary patterns across 163 countries. (A) Scree plot with cumulative variance. (B) PC1 versus PC2 coloured by incidence ASR. (C) PC1 versus PC2 coloured by HDI. PC1 explained 19.3% of the variance and separated animal products, dairy, sugar and fats from rice, cassava and starchy roots; PC2 explained 3.3%. Countries with higher PC1 scores had higher incidence, but PC1 also tracks HDI closely, so the two cannot be separated in this design.

Supplementary Figure S5. PCA loadings for the first two principal components, showing the top 10 positive and negative loading food items for each component. Green bars indicate positive loadings; red bars indicate negative loadings. Dairy, meat, sugar and wheat drive the affluent pattern while rice, sweet potatoes and cassava drive the staple-crop pattern.

Supplementary Figure S6. K-Means dietary clustering. (A) Silhouette scores for k = 2–7. (B) PCA projection by cluster. (C) Incidence by cluster with individual observations. (D) Standardised dietary profiles. (E) Cancer and socioeconomic profiles. (F) Cluster sizes. The optimal solution (k = 2 by silhouette analysis) produced two very unequal groups (n = 150 and 13); the small cluster is the affluent, high-supply group (mean HDI 0.90 versus 0.72) and has much the higher incidence (median ASR 12.1 versus 2.9 per 100,000; Kruskal–Wallis p = 2.08e-06). Because the split is highly imbalanced and the clusters differ in development at least as much as in diet, the incidence gap between them cannot be read as a dietary effect.

Supplementary Figure S7. Observed versus out-of-fold predicted thyroid cancer incidence ASR for the four leading regression models. The red dashed line marks perfect prediction and the green line the linear fit. All models recover the broad gradient but compress it: residuals grow with the observed rate, and the highest-incidence countries — Cyprus and the Republic of Korea in particular — are consistently underpredicted.

Supplementary Figure S8. Classification sensitivity analysis for four representative models. (A) ROC curves with AUC values. (B) Precision–recall curves with AUPRC values. (C) Calibration curves with Brier scores; the dashed diagonal denotes perfect calibration. All curves are computed from pooled out-of-fold predicted probabilities at the median incidence threshold. No conclusion in the manuscript rests on this analysis.

Supplementary Figure S9. Confusion matrices for four classification models (nested stratified 10-fold cross-validation). Each cell shows the number of countries correctly or incorrectly classified as high or low thyroid cancer incidence. Accuracy and F1 scores are displayed above each matrix.

Supplementary Figure S10. Partial dependence plots (PDPs) for the top 8 features from the Gradient Boosting (blue solid line) and Random Forest (red dashed line) models. Shaded bands indicate ±0.5 ASR units. Rug plots at the bottom show the distribution of observed feature values. Partial dependence curves are model summaries evaluated by holding other variables at their observed values; in a dataset where those other variables are strongly correlated with the feature being varied, the curves should not be read as dose–response relationships.

Supplementary Figure S11. Permutation-based feature contribution analysis. (A) Beeswarm plot showing per-country contributions for each of the top 20 features. Each dot represents one country; colour indicates the feature’s standardised value (red = high, blue = low). (B) Mean absolute contribution (bar height) with directional indicators. Green bars indicate features that increase predicted thyroid cancer incidence; red bars indicate features that decrease it. The socioeconomic features dominate the contribution magnitudes.

Supplementary Figure S12. Feature dependence plots for the top 6 features, coloured by development level (blue = HDI > 0.70, orange = HDI ≤ 0.70). The red curve shows a quadratic trend line fitted to all 163 countries. The horizontal dashed line indicates zero contribution. At comparable levels of dietary supply, contributions tend to be larger in higher-HDI countries; because development and diet are so closely tied in these data, this cannot be used to infer an interaction between diet and development.

Supplementary Figure S13. Sensitivity of the seafood–incidence association to the exposure window. (A) Spearman ρ with 2022 incidence for lagged seafood supply at 0, 3, 5, 10 and 12 years. (B) Corresponding −log₁₀ p-values, with reference lines at p = 0.05 and p = 0.001. The correlation varied only between ρ = 0.213 and ρ = 0.232 with no trend towards a stronger association at longer lags. Lagged supply values are highly autocorrelated, so these windows are not independent tests and do not establish temporal precedence; the flatness of the profile is at least as consistent with stable, shared country characteristics — including registry and screening infrastructure — as with a biological latency.

Supplementary Figure S14. Residual diagnostics for one of the leading models (Random Forest), computed on pooled out-of-fold predictions. (A) Residuals versus predicted values. (B) Residual histogram with Shapiro–Wilk test. (C) Normal Q–Q plot. (D) Scale–location plot. Residual normality is not an assumption of these algorithms; the panels are diagnostic rather than inferential. Residuals showed no obvious curvature against predicted values, but they were not symmetric (W = 0.78, p = 1.70e-14) and their spread increased with predicted incidence.

Supplementary Figure S15. Leave-one-region-out validation. (A) Held-out R² and (B) held-out Spearman ρ for each of six world regions and pooled across all regions, for two representative models. Negative R² indicates predictions worse than the training-set mean. Rank agreement is largely preserved while R² is not, indicating that the models transport the ordering of countries across regions but not their level.

Supplementary Figure S16. Sensitivity analyses for the iodine-rich food availability index. Bars are partial correlations with thyroid cancer incidence ASR under the basic adjustment set (HDI, obesity; blue) and the extended adjustment set (adding the three HDI sub-indices and region fixed effects; orange); asterisks mark associations surviving Benjamini–Hochberg correction within each panel (q < 0.05). (A) The three components of the index entered separately in place of the composite: the association is carried by seafood and dairy availability, not by eggs. (B) Four alternative weighting schemes plus a seafood-only variant: the association is insensitive to how the components are weighted, and the equal-weighted and iodine-content-weighted constructions give slightly larger estimates than the primary index. (C) Specificity: an iodine-poor animal-origin food index built from bovine, pig, poultry and mutton and goat meat has the largest unadjusted correlation of any variable examined here but does not survive adjustment, whereas the iodine-rich food availability index does. n = 161 countries with complete socioeconomic data.
